# DNMT1-targeting remodeling global DNA hypomethylation for enhanced tumor suppression and circumvented toxicity in oral squamous cell carcinoma

**DOI:** 10.1186/s12943-024-01993-1

**Published:** 2024-05-16

**Authors:** Yangfan Liu, Yu Sun, Jin Yang, Deyang Wu, Shuang Yu, Junjiang Liu, Tao Hu, Jingjing Luo, Hongmei Zhou

**Affiliations:** 1 State Key Laboratory of Oral Diseases & National Center for Stomatology & National Clinical Research Center for Oral Diseases & Frontier Innovation Center for Dental Medicine Plus, West China Hospital of Stomatology, Sichuan University, Chengdu, 610041 Sichuan China; 2 School of Stomatology, Hainan Medical University, Haikou, 571199 Hainan China

**Keywords:** DNMT1, DNA methylation, Tumor growth, Neoplastic transformation, Oral squamous cell carcinoma, PI3K, Pharmacological toxicity, Insulin feedback

## Abstract

**Background:**

The faithful maintenance of DNA methylation homeostasis indispensably requires DNA methyltransferase 1 (DNMT1) in cancer progression. We previously identified DNMT1 as a potential candidate target for oral squamous cell carcinoma (OSCC). However, how the DNMT1- associated global DNA methylation is exploited to regulate OSCC remains unclear.

**Methods:**

The shRNA-specific DNMT1 knockdown was employed to target DNMT1 on oral cancer cells in vitro, as was the use of DNMT1 inhibitors. A xenografted OSCC mouse model was established to determine the effect on tumor suppression. High-throughput microarrays of DNA methylation, bulk and single-cell RNA sequencing analysis, multiplex immunohistochemistry, functional sphere formation and protein immunoblotting were utilized to explore the molecular mechanism involved. Analysis of human samples revealed associations between DNMT1 expression, global DNA methylation and collaborative molecular signaling with oral malignant transformation.

**Results:**

We investigated DNMT1 expression boosted steadily during oral malignant transformation in human samples, and its inhibition considerably minimized the tumorigenicity in vitro and in a xenografted OSCC model. DNMT1 overexpression was accompanied by the accumulation of cancer-specific DNA hypomethylation during oral carcinogenesis; conversely, DNMT1 knockdown caused atypically extensive genome-wide DNA hypomethylation in cancer cells and xenografted tumors. This novel DNMT1-remodeled DNA hypomethylation pattern hampered the dual activation of PI3K-AKT and CDK2-Rb and inactivated GSK3β collaboratively. When treating OSCC mice, targeting DNMT1 achieved greater anticancer efficacy than the PI3K inhibitor, and reduced the toxicity of blood glucose changes caused by the PI3K inhibitor or combination of PI3K and CDK inhibitors as well as adverse insulin feedback.

**Conclusions:**

Targeting DNMT1 remodels a novel global DNA hypomethylation pattern to facilitate anticancer efficacy and minimize potential toxic effects via balanced signaling synergia. Our study suggests DNMT1 is a crucial gatekeeper regarding OSCC destiny and treatment outcome.

**Supplementary Information:**

The online version contains supplementary material available at 10.1186/s12943-024-01993-1.

## Background

Oral squamous cell carcinoma (OSCC), a heterogeneous malignant tumor originating from oral epithelial cells [1], represents a prototypical form of cancer that undergoes an intricate process of multistage carcinogenesis, encompassing epithelial hyperplasia, dysplasia and carcinoma in situ [2]. Due to its tendency for recurrence and metastasis following treatment, OSCC patients, especially those in advanced stages, exhibit a low survival rate [3]. To improve the survival outcome of OSCC patients, novel therapeutic approaches have been devised to increase the survival time of OSCC patients by selectively targeting aberrant signaling molecules or proteins in cancerous cells, such as EGFR and PD-L1 [4–6]. Nevertheless, the efficacy of these targeted therapies is often hindered by the emergence of agent resistance in cancer cells, which is attributed to various factors including genomic instability, dysregulation of signal cascades or pharmacological toxicity [7, 8]. Thus, it’s imperative to gain a comprehensive understanding of the underlying mechanism that governs epithelial carcinogenesis and tumor growth, with the aim of identifying potential therapeutic targets for the prevention and treatment of OSCC.

DNA methylation, a prevalent epigenetic modification that governs gene expression patterns, cell type-specific genome stability and embryonic development in eukaryotes [9], has been closely linked to oral cancer progression [10, 11]. Changes in DNA methylation patterns associated with carcinogenesis progress gradually with cell proliferation. Notably, cancer cells possess a genome-wide DNA hypomethylation landscape, contributing to cancer cell instability and tumor heterogeneity [12]. DNA methyltransferase 1 (DNMT1), the most prevalent DNA methyltransferase, is crucial for maintaining DNA methylation homeostasis during DNA replication [13]. Any alteration in DNMT1 stability and activity can result in extensive changes of DNA methylation [14–17]. Our earlier study predicted DNMT1 as a potential target associated with OSCC progression [18]. Previous studies have shown that DNMT1 inhibition has positive anticancer effects on various types of squamous cancers [19–21]. However, the mechanism by which DNMT1 regulates OSCC initiation and progression via DNA methylation and signal transduction remains largely unknown.

Several DNA demethylating agents have undergone clinical trials to treat malignant tumors including head and neck cancers, yet individual treatment outcomes are variable and are being investigated further [22]. Cancers harbor intricate signaling networks that regulate numerous cellular processes, including proliferation, cell death and metabolism; these internal factors influence targeted agents. This aforementioned interactive network is of paramount importance in facilitating the indefinite proliferation and sustained viability of cancer cells [23–25]. Thus, deliberate interference with essential signaling pathways that result in cell growth arrest or death, such as PI3K and CDK inhibitors, is widely regarded as a significant approach for preventing neoplastic transformation and eradicating cancer cells [26–28], although some of the aforementioned limitations remain. Considering that DNA methylation is viewed as an upstream regulator of signal transduction modifications [29], DNMT1, which plays an indispensable role in maintaining genome-wide DNA methylation status, could act as a gatekeeper to maintain the equilibrium of dynamic signal transduction.

In this study, our objective was to elucidate the regulatory role of the DNMT1- dependent global DNA hypomethylation pattern in controlling OSCC development. To achieve this goal, we integrated multilayered experimental data from clinical human samples, xenograft mouse models, and independent high-throughput microarray analysis of DNA methylation alongside bulk and single-cell transcriptome analysis. We demonstrated that DNMT1-knockdown remodels sheer genome-wide DNA hypomethylation in OSCC cells. This novel DNMTI-specific DNA hypomethylation pattern triggers dual inhibition of PI3K-AKT and CDK2-Rb and inactivation of GSK3β, leading to enhanced tumor regression in comparison to that of a PI3K inhibitor. GSK3β deactivation also counteracts the adverse pharmacological toxicity of hyperglycemia and insulin feedback caused by PI3K inhibition. These combined effects create a synergistic function of signal transduction, ultimately resulting in enhanced efficacy and reduced toxicity in treating OSCC. Our research suggested that DNMT1 can serve as an essential gatekeeper of multiple signals, rendering it a promising target for controlling oral neoplastic transformation and improving OSCC treatment.

## Materials and methods

### Patient tissue samples

Human OSCC tissues (*n* = 22) were obtained from resected primary tumors without recurrence, and without prior chemotherapy or radiotherapy. Human hyperplasia tissues (*n* = 6) and dysplasia tissues (*n* = 7) were collected from bioptic oral leukoplakia lesions without any prior treatment. Normal human tissues (*n* = 15) were procured from healthy oral mucosa during orthognathic surgery or wisdom tooth extraction. All tissue harvesting procedures were performed at West China Hospital of Stomatology, Sichuan University, and approved by the Human Research Ethics Committee of West China Hospital of Stomatology, Sichuan University (No. WCHSIRB-D-2021–548). The tissue samples were fixed in 10% neutral formalin for 24 h, transferred to 75% alcohol and embedded in paraffin. Pathological examination confirmed the identity of all the samples.

### Cell culture and cell lines

The Cal27 and FaDu cell lines were obtained from the ATCC (United States); the HSC-3 cell line was obtained from the JCRB Cell Bank (Japanese); the SCC4 cell line was purchased from the BeNa Culture Collection (BNCC, China); and the UM-SCC1 and human normal oral keratinocyte (NOK) cell lines were obtained from the State Key Laboratory of Oral Diseases. All cancer cell lines were authenticated by STR analysis. Cal27, FaDu, SCC4, UM-SCC1 and HSC-3 cells were cultured in DMEM (Gibco, Grand Island, NY, USA) supplemented with 10% fetal bovine serum, while NOK cells were cultured in defined keratinocyte SFM (Gibco, Grand Island, NY, USA) with corresponding growth supplement. All cells were maintained at 37 °C in a humidified atmosphere with 5% CO_2_. For cellular intervention involving inhibitors or activators, all reagents were applied at specific final concentrations, which were diluted with dimethyl sulfoxide (DMSO). An equivalent volume of DMSO was added as a negative control. The final concentrations are indicated as described in the figure legends.

### Antibodies and reagents

Antibodies against DNMT1 (EM1901-83), BrdU (RT1081), mTOR (ET1608-5), GSK3β (ET1607-71), p-GSK3β (Ser9, ET1607-60) and PKM2 (ER1802-70) were obtained from HuaBio. Antibodies against Ki67 (9449 T, for immunostaining), PI3K (4249 T), p-mTOR (Ser2448, 5536 T), AKT (4691 T), p-AKT (Ser473, 4060 T), cleaved caspase-3 (CC3, 9664S) and p-Rb (Ser807/811, 8516S) were obtained from Cell Signaling Technology. Anti-Ki67 antibody (ab16667, for immunoblotting), anti-CDK1 (phospho T161) + CDK2/CDK3 (phospho T160) antibody (ab201008) and anti-phosphofructokinase antibody (PFK, ab154804) were obtained from Abcam, while an antibody against 5-mC (A-1014) from Epigentek. All the inhibitors and activators used were from MCE, including BEZ235 (HY-50673) for PI3K inhibition, 740Y-P (HY-P0175) for PI3K activation, AT7519 (HY-50943) for CDK2 inhibition, and GSK-3484862 (HY-135146) and GSK-3685032 (HY-139664) for DNMT1 inhibition.

### Immunoblotting

Total cell proteins were extracted in lysis buffer (SAB signalway antibody, USA). After gel electrophoresis, the proteins were transferred to PVDF membranes (BioRad, Hercules, CA, USA) and blocked with 5% BSA solution. The blots were incubated with primary antibodies (as described above) at 4℃ overnight, with GAPDH or β-Tubulin serving as the control. Then, the membranes were incubated with the appropriate secondary antibodies (SAB signalway antibody, USA) for 1 h at room temperature, and signal detection was then performed using an Easy ECL protein blotting reagent kit (TransGen Biotech, China), and image scanning was performed using a Chemidoc XRS imaging system (BioRad, Hercules, CA, USA). Blotting images are representative from 3 repeats at least.

### Lentiviral construction and cell transfection

DNMT1 was silenced by short-hairpin RNA using the targeting sequences 5'ACTACATCAAAGGCAGCAA-3' (sh-DNMT1-1) and 5'GGATGAGTCCATCAAGGAAGA-3' (sh-DNMT1-2). A scrambled control sequence was used as a control (sh-NC). All recombinant lentiviral viruses were purchased from Shanghai Genechem Co., Ltd. (https://www.genechem.com.cn/), and generated using the hU6-MCS-CBh-gcGFP-IRES-puromycin vector and GV493 packaging cells. Then, Cal27 and FaDu cells were transfected following the manufacturer's instructions, and harvested after 72 h of transfection after selection with puromycin.

### Cellular immunofluorescence

Immunofluorescence staining of cells was performed as previously described [30]. Briefly, cells were fixed with 10% neutralized formalin followed by permeabilization with 0.3% Triton X-100. For anti-BrdU staining, cells were first incubated with 10 µM BrdU-labeled culture medium for 6 h before cell fixation, followed by antigen retrieval with 1 M HCl. After washing with PBS, the cells were blocked with 10% goat serum for 30 min. Then, primary antibodies (as deacribed above) were applied, followed by incubation with the appropriate secondary antibodies and nuclear DAPI staining. Images were captured using an inverted fluorescence microscope (Leica, Germany).

### Sphere formation assay

The medium for the sphere formation assay was prepared by adding 1 × B27 (Corning), 20 ng/ml EGF (PeproTech) and 10 ng/ml bFGF (PeproTech) to DMEM/F12. The cancer cells were resuspended in the above medium at an adjusted density of 2000 cells/ml and then inoculated on ultralow adhesion 24-well plates with 500 μl of cell suspension per well. A volume of 200 μl of fresh medium was added to each well every 2 days. Images were captured 10 days after cell seeding using an inverted fluorescence microscope (Leica, Germany).

### OSCC xenograft model and animal administration

All animal experiments were reviewed and approved by the Animal Care and Use Ethics Committee of West China Hospital of Stomatology, Sichuan University (No. WCHSIRB-D-2021–628). Six- to eight-week-old female BALB/c nude mice (Beijing Vital River Laboratory Animal Technology Co., Ltd., China) were used as tumor recipients. As described previously [31], a total of 3 ~ 5 × 10^6^ viable Cal27 cells WT, sh-NC and sh-DNMT1 cells with 50% Matrigel (Corning, USA) were subcutaneously transplanted into the right flank of anesthetized mice. All viable cells were confirmed and quantified using an automatic Cell Counter (Countess3, Invitrogen) with Trypan blue staining. When the tumor started to grow, the tumor volume was measured every other day and calculated using the formula 0.50 × long axis × short axis^2^. Beginning at the second or third week (depending on the cell density of the primary injection), after transplantation, when the xenografted tumor reached to at least 200 mm^3^, the mice were rearranged according to body weight and tumor volume, and the intervention started.

For PI3K inhibitor treatment, mice were treated with BEZ235 (Dactolisib, 10 mg/kg/day) by oral gavage or an equal volume of vehicle (10% 1-Methyl-2-pyrrolidinone and 90% Poly) daily for 7–14 consecutive days before being sacrificed, after which the tumors were harvested. For PI3K activator or CDK inhibitor administration, mice were treated with 740Y-P (20 mg/kg/day) or AT7519 (15 mg/kg/day) by intraperitoneal injection (i.p.) or an equal volume of vehicle (2% DMSO + 30% PEG 300 + 2% Tween 80 + ddH_2_O) daily for 7 or 14 days respectively according to the same treatment cycle of PI3K inhibitor treatment. For combined intervention, a CDK inhibitor was applied to mice 30 min after PI3K inhibition. BrdU (100 mg/kg) was administered i.p. 2 h before the tumors were harvested.

### Histopathology, immunohistochemistry (IHC), immunofluorescence (IF), terminal deoxynucleotidyl transferase dUTP nick end labeling (TUNEL) assay, and periodic acid-Schiff (PAS) staining

Human tissues, and subcutaneous tumors harvested at the endpoint of the study were paraffin-embedded, sectioned, and stained with hematoxylin and eosin (H&E) for histopathological diagnosis. Then, IHC and IF staining were performed as previously described [32]. Briefly, after dehydration, rehydration and antigen retrieval, paraffin-embedded sections (pretreated with 3% H_2_O_2_ for IHC) were blocked with 15% normal goat serum (NGS) at room temperature for 1 h and then incubated with primary antibodies at 4 °C overnight. For IHC, secondary antibodies conjugated to HPR were used following the addition of brown DAB substrate for target staining and hematoxylin for nuclear staining. For IF, secondary antibodies conjugated to Alexa Fluor 594 (red) or 488 (green) were used (1:500 for all; Cell Signaling Technology), followed by DAPI (Beyotime, China) for nuclear staining. Terminal deoxynucleotidyl transferase dUTP nick end labeling (TUNEL) staining was performed with a TMR (red) TUNEL Cell Apoptosis Detection Kit (Solarbio, China) according to the manufacturer's directions to detect apoptotic cells. PAS staining was performed with an AB-PAS Stain Kit with hematoxylin (Solarbio, China) to detect glycogen according to the manufacturer's directions. All slides were mounted with coverslips using Fluoromount-G (Southern Biotech).

### Bioinformatics analysis

The differential pancancer mRNA expression of DNMT1 was analyzed by the Sangerbox platform [33]. The bulk analysis was performed with the GSE30784 dataset, which includes 45 normal tissue, 17 dysplasia, and 167 OSCC tissues, as well as the DNMT1 RNA-seq datasets of OSCC, which include OSCC tissues (*n* = 350) and normal tissues (*n* = 15) adjacent to cancer, and pancancer analysis in The Cancer Genome Atlas (TCGA) database.

The global DNA methylation data, normalized as total β values log2, were downloaded from GSE204943. This dataset comprised samples from normal tissues (*n* = 22), oral leukoplakia (OLK) tissues (*n* = 22), typical oral precancerous lesions with hyperplasia or dysplasia, and OSCC tissues (*n* = 74), detected using 850 k Infinium Methylation EPIC BeadChips (850 k chip). Additionally, OSCC data from the TCGA database were utilized. For the GSE204943 dataset, we applied the ChAMP package to, filter the data, perform quality control, normalize the data and perform differential methylation site (DMS) analysis. We depicted the distribution of normalized β values and conducted principal component analysis (PCA) to visualize the sample distribution across the three groups. To determine the quality of the methylation data among the three groups, we sorted the variance of each row in the matrix from smallest to largest, and selected the top 1000 rows. The multidimensional scaling (MDS) graph and heatmap were then used to display these 1000 most variable positions.

For both DNMT1 RNA-seq data and global DNA methylation data (normalized as total β values log2) downloaded from the TCGA database, by using Empower (R) software, a smooth curve fitting was carried out respectively based on the clinicopathological data in TCGA database, omitting patients who had radiotherapy and chemotherapy, and adding the follow-up time. Then, data processing and analysis of Kaplan–Meier and univariate/multivariable Cox-regression were performed using R version 4.3.0 (2023–04-21), along with Storm Statistical Platform (www.medsta.cn/software) and GraphPad Prism 9.4. The R4.2.2 package was used for restricted cubic spline analysis of hazard ratio in overall survival and ggplot2 for visualization.

### High-throughput DNA methylation microarray

To detect the global DNA methylation level of cells, we used an 850 k chip by Shanghai Biotechnology Corporation. Cell pellets of NOK and Cal27 cells, including nontransfected (referred to as WT), sh-NC, and sh-DNMT1 cells, were collected at a density of 1 × 10^7^ cells per group with at least three independent replicates for each cell line. After DNA extraction, the Qubit Fluorometer method was used to quantify the DNA samples, while agarose gel electrophoresis and bisulfite conversion were used for quality inspection. Then, whole-genome amplification (WGA), fragmentation, alkaline denaturation, chip hybridization, washing, extension, imaging, and scanning were conducted to obtain the raw data of the chip. All the raw Illumina methylation microarray data were converted to methylation values (beta values, β) and then normalized within-array using the subset-quantile within array normalization (SWAN) algorithm. DMSs between groups were calculated based on β values, using the pool.t-test method, setting the threshold as *P* < 0.05, |beta. difference|> 0.1. Information on the differentially methylated sites included the *P* value, adjusted *P* value (FDR), and beta value. Differences and annotation information. A clustering heatmap of the differentially methylated sites was generated with Sangerbox (http://vip.sangerbox.com/login.html). ChAMP was used for the DMS analysis of key genes (R package ChAMP v2.30.0 and DMP.GUI function). All the original EPIC microarray data have been deposited in GEO with the accession ID GSE262310.

### RNA extraction and sequencing

Total RNA was extracted using TRIzol reagent (R411-C3, Vazyme, China) following the manufacturer’s protocol. Three replicates of RNA samples from each cell type were used for cDNA library construction. RNA purity and quantification were determined using a NanoDrop 2000 spectrophotometer (Thermo Scientific, USA), while RNA integrity was assessed using an Agilent 2100 Bioanalyzer (Agilent Technologies, Santa Clara, CA, USA). Transcriptome libraries were then constructed following the instructions provided with the VAHTS Universal V5 RNA-seq Library Prep Kit. Finally, the RNA libraries were sequenced with an Illumina NovaSeq 6000 system by OE Biotech Co., Ltd. (Shanghai, China). All the original RNA-seq data have been deposited in GEO with the accession ID GSE262505.

### Screening of key functional genes downstream of DNMT1-DNA methylation

Four available DNA methylation datasets (GSE123781, GSE87053, GSE75537, and GSE136704) were ultimately obtained from the Gene Expression Omnibus (GEO). These datasets met specific criteria including pathological confirmation for OSCC, DNA methylation data from the same detection platform, normal oral tissues as controls, and patients who had not received chemo-/radiotherapy or medical therapy. The four available methylation GEO datasets and the TCGA methylation dataset above were combined. The combined datasets were standardized using the minfi package. Then, differentially expressed CpG sites were screened using the R packet ChAMP. To identify consistency across datasets, the Meta R package was further used for the aforementioned differential CpGs. With a single CpG |logFC|> 0.1 and a false discovery rate (FDR) < 0.05 as the thresholds, all the meta- *P* values were further corrected by the Benjamini–Hochberg method to identify the final differential CpGs and their differential DNA methylation genes (DMGs).

Moreover, after normalizing the original TCGA RNA-seq data, the genes coexpressed with DNMT1 were obtained by Pearson correlation analysis (r), with correlation coefficients of r ≥ 0.3 and *P* < 0.05 as filtering conditions. Next, using Cor ≥ 0.4 and *P* < 0.05 as the threshold, differential expression genes (DEGs) that interact with DNMT1 were identified. Subsequently, we overlapped these DEGs with the identified DMGs above, and the DMGs associated with the top 20,000 differential CpG sites from sh-DNMT1 vs sh-NC OSCC cells, obtained from our DNA methylation high-throughput microarray analysis. This intersection yielded 152 genes that exhibited dual correlations with both DNMT1 and DNA methylation. Next, we conducted PPI network analysis using the String website, and visualized the network using Cytoscape 3.6.0. Kyoto Encyclopedia of Genes and Genomes (KEGG) enrichment analysis was performed to identify potential functional pathways.

Moreover, FunRich3.1.3 was used to construct a PPI network filter for these 152 screened genes. With Nodes ≥ 100 as the standard, the 5 key node genes closely associated with DNMT1-DNA methylation were screened. Finally, based on the TCGA data and considering that the RNA expression of these genes was significantly different from that in normal tissues, we identified the key functional genes downstream of DNMT1-DNA methylation (*P* < 0.05).

### Gene function enrichment analysis

All the differentially methylated sites located in CpG islands were sorted in descending order based on |beta. Difference|, and the corresponding genes were extracted. After removing all blank values and duplicates, the top 3000 genes were selected for subsequent gene function enrichment analysis.

For Gene Ontology (GO) biological process enrichment analysis, the subset (c5.go.bp.v7.4.symbols.gmt) was downloaded from the Molecular Signatures Database (http://www.gsea-msigdb.org/gsea/downloads.jsp) [34], as the background gene set to map genes. For KEGG enrichment analysis, the most recent gene annotations were obtained from the KEGG REST API (https://www.kegg.jp/kegg/rest/keggapi.html), as the background gene set to map genes. Next, the R software package clusterProfiler (v3.14.3) was used for both GO and KEGG enrichment analysis, with a threshold setting of 5 to 5000 for the gene set, *P* < 0.05 and FDR < 0.1.

### Multiplex immunohistochemistry (mIHC) staining

For the mIHC staining in this study, a multicolor-kit (Absin, China) was used as previously described [35]. We used the Opal 520 channel [fluorescein isothiocyanate (FITC), a green fluorescence stain], the Opal 570 channel [cyanine 3 (Cy3), an orange fluorescence stain], and the Opal 670 channel [cyanine 5 (Cy5), a red fluorescence stain] to locate different proteins (as detailed in the figure legends). DAPI was used for nuclear staining. All the slides were observed and imaged with an Olympus FV1200 confocal microscope (Tokyo, Japan).

### Glucose and insulin measurement

For xenografted mice treated with vehicle, BEZ235 and/or AT7519, peripheral blood was collected from the tail of mice every 60 min and subjected to glucose measurement with a Rapid Blood Glucose Monitor (Sinocare, China). Blood insulin was measured 5 h after mouse administration with an Ultra Sensitive Mouse Insulin ELISA Kit (Crystal Chem).

### Single-cell transcriptome analysis

The single-cell RNA sequencing (scRNA-seq) dataset GSE181919 was downloaded [36], and included 9 normal tissue samples, 4 precancerous leukoplakia samples and 20 primary HNSCC samples. The QC process was performed using Seurat (v4.3.0.1) in R version 4.3.1. The Seurat anchor-based integration method was used to correct the batch and merge 33 samples. Low-quality cells with fewer than 200 or more than 7000 unique molecular identifiers (UMIs) or more than 30% mitochondrion-derived UMI counts were filtered out. From these, we obtained a final dataset of 45,754 single-cell transcriptomes, following normalization using the NormalizeData and ScaleData functions. The top 20 principal components along with the top 2000 variable genes were used in this process. Dimensionality reduction through principal component analysis reduced variables and finally clustered the cells. The main cell clusters were identified using the FindNeighbors and FindClusters functions (dims = 40, resolutio* n* = 0.1) of Seurat and visualized using t-distributed stochastic neighbor embedding (tSNE). Cell-type annotation was performed by the R package SingleR (v2.3.5), alongside manual comparison of marker gene expression across different clusters by the FindAllMarkers function. A particular set of marker genes was projected into dot plots for cell type identification. To accurately distinguish cancer cells from epithelial cell clusters, the R package CopyKAT v1.1.0 was used to classify epithelial cells as either aneuploid (to represent malignant cells) or diploid [37]. Finally, 5258 single epithelial cells, including 1562 aneuploid cells and 3696 diploid cells, were subjected to pseudotime trajectory analysis.

To reveal the changes in epithelial cells during the multistage carcinogenesis process, we used Monocle 2 (v2.29.0) [38]. The following parameters were set as mean expression ≥ 0.1 and qval < 0.01 via the differential GeneTest function. The trajectories were visualized by the plot_pseudotime_heatmap and plot_complex_cell_trajectory functions, as well as the geom_density function in the R package ggplot2 v3.4.2. To calculate pathway activity for each epithelial cell, the R package AUCell (v1.23.0) and AddModuleScore_UCell function were utilized to calculate the hallmark gene set score, and ggplot2 was subsequently used for visualization.

### Statistical analyses

The normality of the group datasets was assessed using the Shapiro–Wilk normality test. Statistical differences between two groups were analyzed using either an unpaired Student’s t test or a nonparametric Mann–Whitney exact test, depending on the data distribution. For comparisons involving more than two groups, one-way ANOVA with Tukey’s multiple comparison test was used. All the statistical analyses were conducted using GraphPad Prism version 9 (GraphPad Software, San Diego, CA, United States). Individual data points represent values obtained from technical or biological replicates.

## Results

### DNMT1 overexpression gradually increases during oral neoplastic transformation and is linked to tumor growth in OSCC

**Fig. 1:**
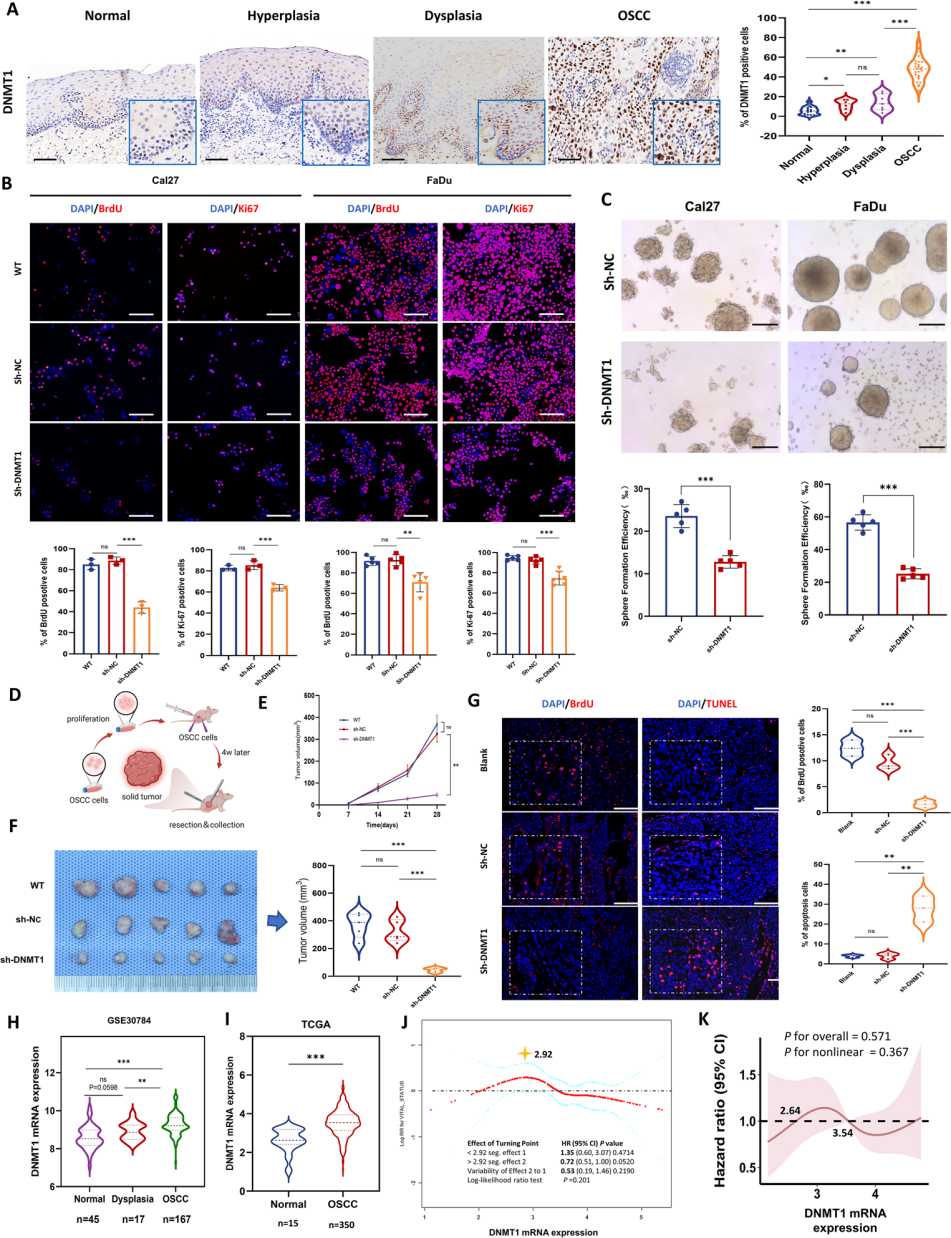
DNMT1 expression increases along with oral neoplastic transformation and its overexpression is correlated with tumor growth. **A** Representative IHC images and analysis of DNMT1 in oral human samples including normal (*n* = 15), hyperplastic (*n* = 6), dysplastic (*n* = 7) and OSCC tissues (*n* = 22). Scale bars, 100 μm. **B** Immunofluorescence of BrdU and Ki67 in OSCC cell lines. The data are presented as the means ± SDs of three independent experiments. Scale bars, 100 μm. **C** Sphere formation assay and statistical quantification of OSCC cell lines. Scale bars, 100 μm. **D** Schematic showing the xenografted OSCC mouse model. *n* = 5 mice in each group. **E** Tumor growth curve. **F** Presentation of OSCC xenografted tumors and tumor volume statistics at the endpoint of the study. **G** Immunofluorescence and analysis of BrdU and TUNEL in OSCC xenografted tumors. Scale bars, 100 μm. **H** The mRNA expression of DNMT1 in multiple stages of carcinogenesis, including normal (*n* = 45), oral dysplasia (*n* = 17), and OSCC (*n* = 167) (data provided by GSE30784). **I** The mRNA expression of DNMT1 in OSCC (*n* = 350) compared to that in oral normal tissues (*n* = 15) (data from TCGA). **J** Smooth curve fitting showing the correlation between DNMT1 expression and mortality risk in OSCC patients. **K** Restricted cubic spline analysis indicating the correlation of DNMT1 expression with the hazard ratio of overall survival in OSCC patients. The values of DNMT1 RNA expression were 2.64 and 3.54, respectively, when HR = 1. *P < 0.05, **P < 0.01, and ***P < 0.001 according to unpaired Student’s* t* test or one-way ANOVA with Tukey’s multiple comparison test

To evaluate the expression of DNMT1 during OSCC development, we examined human samples ranging from oral normal tissue to hyperplastic and dysplastic lesions, and ultimately to OSCC tissues. The results revealed a progressive increase in DNMT1 expression with the process of oral neoplastic transformation, peaking in OSCC tissues (Fig. 1A). A series of OSCC cell lines were utilized and initially confirmed to have markedly higher DNMT1 expression than NOK cells (Fig. S1A). Representative Cal27 and FaDu cells were then used to generate DNMT1-knockdown cell lines (sh-DNMT1-1 and sh-DNMT1-2) (Fig. S1B and C). Given the more pronounced gene silencing effect of sh-DNMT1-1, it was selected for subsequent investigations. DNMT1 silencing led to a considerable reduction in cell proliferation, as evidenced by decreases in BrdU and Ki67 expression (Fig. 1B) and in the sphere-forming capacity of OSCC cells (Fig. 1C) in vitro. Furthermore, an OSCC xenograft mouse model was generated (Fig. 1D). sh-DNMT1-transfected cancer cells exhibited a notably reduced tumorigenic ability, as indicated by decreased tumor growth, reduced cell proliferation and augmented cell apoptosis (Fig. 1, E–G; Fig. S1D).

Utilizing the RNA-seq data of the GSE30784 dataset (Fig. S2, A-C), we observed a gradual increase in DNMT1 expression during oral neoplastic transformation (Fig. 1H). Subsequently, we procured primary OSCC and normal tissues from the TCGA database, and conducted further analysis on a larger patient cohort with matched clinicopathological and follow-up records. DNMT1 was overexpressed in OSCC tissues compared to adjacent normal tissues (Fig. 1I). Smoothing curve fitting using clinicopathological and follow-up data from patients revealed a nonlinear and dynamic association between DNMT1 expression and the risk of OSCC mortality, although the difference was not significant (Fig. 1J). Briefly, there was a general upward trend in mortality risk with increasing DNMT1 expression before the infection point at 2.92; thereafter, the potential relationship reversed. In addition to linear analyses such as multivariate Cox regression or Kaplan ‒ Meier analysis (Fig. S1E and F), we opted to assess their nonlinear relationship using restricted cubic spline analysis. This analysis demonstrated a fluctuating relationship between DNMT1 expression and the hazard ratio of overall survival (Fig. 1K). Notably, a significant decrease in DNMT1 expression in cancer correlated with a notable reduction in mortality and the hazard ratio for overall survival. Pancancer analysis confirmed DNMT1 overexpression in various cancer types, including head and neck cancers (Fig. S2D), indirectly suggesting that DNMT1 is a potential target. Our findings indicate that DNMT1 overexpression contributes to the initiation of oral malignant transformation and facilitates the malignant behavior of cancer cells, resulting in the growth of OSCC tumors.

**Fig. 2:**
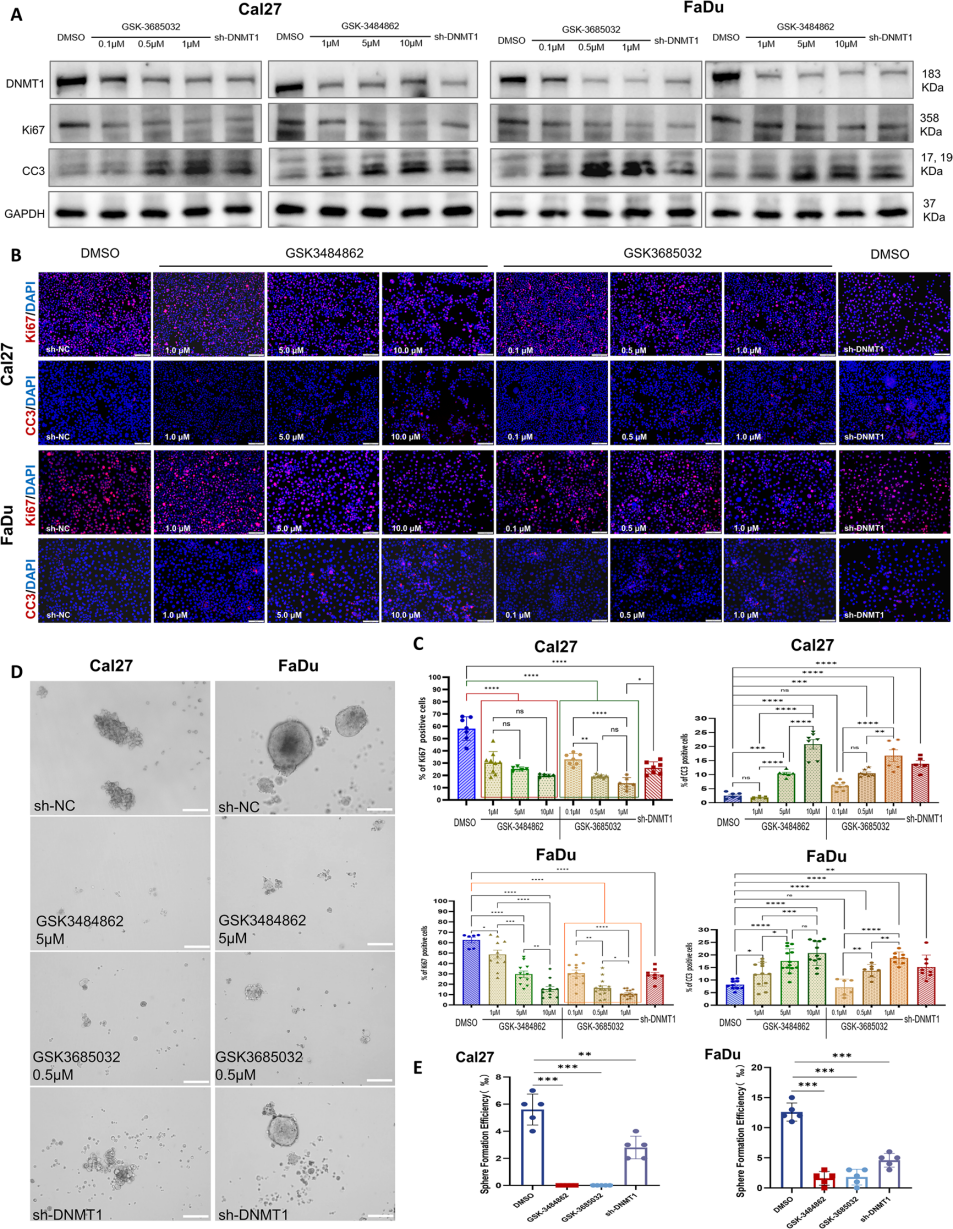
DNMT1 inhibitors consistently significantly inhibited proliferation and promoted of apoptosis in OSCC cells. **A** Western blot analysis of DNMT1, Ki67, and CC3 in OSCC cells. GSK-3484862 and GSK-3685032 were diluted in DMSO at different concentrations. The data are shown as the mean ± SEM. **B** and** C** Immunofluorescence and statistical quantification of Ki67 and CC3 in OSCC cells. Scale bars, 100 μm.** D** and** E** Sphere formation assay and statistical quantification of OSCC cells. Scale bars, 100 μm. *P < 0.05, **P < 0.01, ***P < 0.001, and ****P < 0.0001 by one-way ANOVA with Tukey’s multiple comparison test

To further validate the potential of DNMT1 to target OSCC, we selected established DNMT1 inhibitors, namely, GSK-3484862 and GSK-3685032 [39, 40], for parallel experiments to determine the role of DNMT1 inhibition in the biological behaviors of OSCC cells, primarily proliferation and apoptosis. After one week of uninterrupted intervention, both inhibitors consistently reduced DNMT1 expression in OSCC cells (Fig. 2A). At various concentrations, both inhibitors decreased the expression of the proliferation marker Ki67 and increased the expression of the apoptosis marker cleaved caspase 3 (CC3) in OSCC cells, consistent with the effects of DNMT1 silencing (Fig. 2 A-C). Functionally, both inhibitors effectively suppressed the self-renewal capacity of OSCC cells (Fig. 2 D and E). These results further reinforce the notion that DNMT1 is a promising target for inhibiting the malignant behaviors of OSCC cells.

### Genome-wide DNA hypomethylation occurs during oral carcinogenesis and is stably maintained as a cancer-specific homeostasis in OSCC

**Fig. 3:**
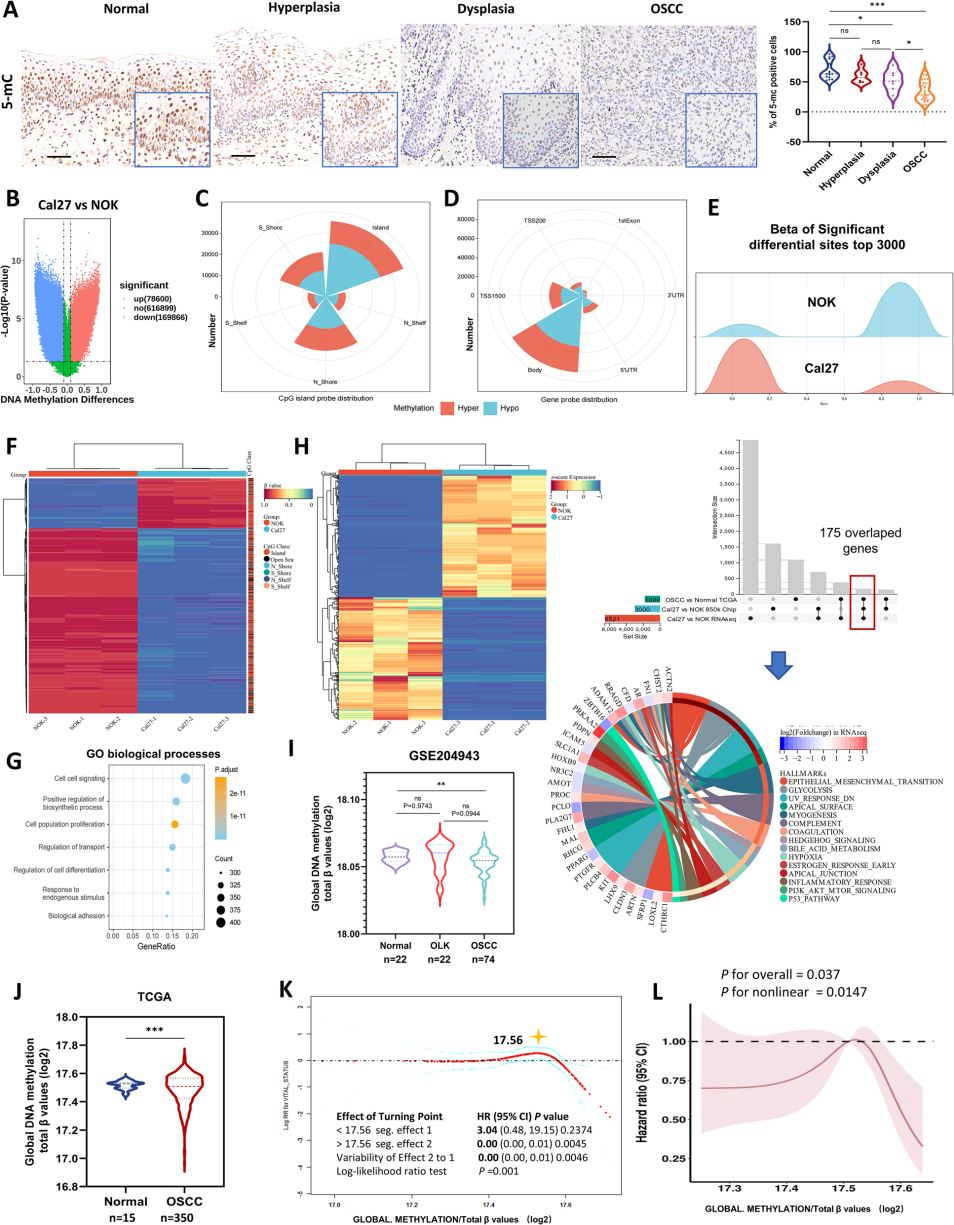
Global DNA hypomethylation occurs during oral carcinogenesis and is relatively stable in cancer cells and is associated with OSCC prognosis.** A** Representative IHC images and analysis of 5-mC in oral human samples, including normal (*n* = 15), hyperplastic (*n* = 6), dysplastic (*n* = 7) and OSCC (*n* = 22) tissue samples. Scale bars, 100 μm. **B** Volcano plots from the DNA methylation 850 k chip showing significantly differential CpG sites in Cal27 cells compared to NOK cells. **C** and** D** Nightingale rose chart showing the number of all significant DMSs with a CpG island probe distribution (C) and a gene probe distribution (D), respectively. **E** Ridge plot showing the β value distribution of the top 3000 significant differential sites in Cal27 cells and NOK cells. **F** Heatmap showing the top 1000 differential CpG sites with the absolute differences in β values. The class of CpGs (in relation to CpG islands) is shown on the right of the heatmap. **G** Bubble chart showing GO biological process enrichment of genes related to differential DNA methylation sites. The differential DNA methylation sites distributed on CpG islands were sorted by β value, and the top 3000 related genes were selected for enrichment analysis. **H** Heatmap showing the top 500 upregulated genes and 500 downregulated genes in Cal27 and NOK cells according to RNA-seq. UpSetR visualized overlapping genes among the DMGs and DEGs of Cal27 and NOK, as well as DEGs from the TCGA database. The bottom circle graph shows the hallmark gene set enrichment of the 175 overlapping genes. **I** Global DNA methylation (normalized as total β values log2) in OSCC (*n* = 74) compared with that in OLK tissues (typical oral precancerous lesion, *n* = 22) and normal oral tissues (*n* = 22) based on GSE204943 dataset. **J** Global DNA methylation (normalized as total β values log2) in OSCC (*n* = 350) compared with that in normal oral tissues (*n* = 15) based on TCGA data. **K** Smooth curve fitting showing the correlation between global DNA methylation and mortality risk in OSCC patients. **L** Restricted cubic spline analysis indicating the correlation of global DNA methylation with the hazard ratio of overall survival in OSCC patients. *P < 0.05, **P < 0.01, and ***P < 0.001 by unpaired Student’s* t* test or one-way ANOVA with Tukey’s multiple comparison test

DNMT1 alteration directly induces changes in genome-wide DNA methylation [41]. Thus, we examined DNA methylation in a set of freshly collected human samples comprising oral normal tissue, hyperplastic and dysplastic lesions, and OSCC tissues, by detecting 5-methylcytosine (5-mC), a covalent methylation at the fifth carbon atom of cytosine that is acknowledged as a specific marker of global DNA methylation [42]. Notably, both dysplastic and OSCC tissues showed significantly lower 5-mC expression than normal tissues, while there was no significant difference between normal and hyperplastic tissues (Fig. 3A).

To delve deeper into genome-wide DNA hypomethylation, we employed 850 k chip to determine the global DNA methylation status of OSCC cells (Fig. S 3, A and B). Compared to normal cells, OSCC cells exhibited prominently differential DNA hypomethylation sites, with an approximately 2.16-fold count of hypermethylated sites (Fig. 3B). The distribution of differential CpG sites between these two cell types mirrored that of the Infinium probe distribution on the 850 k microarray (Fig. 3, C and D; Fig. S 3, C and D). Most DMSs were located within CpG islands rather than in the adjacent island shores, the region within 2 kb of the islands (Fig. 3C; Fig. S 3D). When employing gene region probes, the majority of DMSs were placed within the gene body region rather than the promoter (Fig. 3D; Fig. S 3D). Analysis of the β values of the top 3000 significant DMSs revealed greater hypomethylation than in NOK cells (Fig. 3E). Analysis of the top 1000 significant DMSs indicated that a small portion of significant hypermethylation sites occurred in OSCC cells, with most of them located within CpG islands (Fig. 3F). Together, these DNA methylation microarray data indicated a cancer- specific hypomethylation status in OSCC cells. This internal hypomethylated homeostasis may be related to the maintenance of cancer cell survival, as evidenced by the enrichment of biological processes, including cell–cell signaling, cell population proliferation and regulation of transport and cell differentiation through GO analysis (Fig. 3G; Fig. S 3E), and the involvement of signal transduction pathways, such as the PI3K-AKT, MAKP, and Rap1 signaling pathways (Fig. S 3E), according to KEGG classification.

To better figure out the potential functions of these DMGs, additional RNA-seq of Cal27 and NOK cells was performed (Fig. S 3F), revealing 979 upregulated genes and 629 downregulated genes with |logFC|≥ 1 and FDR < 0.05 (Fig. S 3G). When overlapping these DMGs from 850k chip, DEGs from both cell RNA-seq and TCGA database, the hallmark gene set enrichment analysis identified pathways closely linked to epithelial malignancy and cancer progression, including Hedgehog, Hypoxia, PI3K-AKT-mTOR, P53 and epithelial- mesenchymal- transition (Fig. 3H).

In addition, we analyzed global DNA methylation using the GSE204943 dataset, encompassing data from normal tissues, OLK lesions and OSCC tissues (Fig. S 4). While DNA methylation levels in OLK lesions did not decrease compared to those in normal samples, a significant reduction was observed in OSCC tissues (Fig. 3I). This could be because the OLK lesions in this dataset were not pathologically subclassified as hyperplasia or dysplasia. Further analysis of the TCGA database confirmed lower global DNA methylation in OSCC tissues than in adjacent normal tissues (Fig. 3J). Also, smoothing curve fitting revealed a nonlinear and dynamic association between global DNA methylation and mortality risk, with a single inflection point at 17.56 (normalized by log2), corresponding to a total β value of 193,145.44. A higher level of DNA methylation was associated with a lower mortality risk in OSCC patients (Fig. 3K). A nonlinear association between the hazard ratio and global DNA methylation was observed, with a decrease in the hazard ratio with a substantial alteration in overall DNA methylation, either through a significant decrease or increase, leading to an improved survival rate (Fig. 3L).

Taken together, these findings strongly suggest that the progressive genome-wide hypomethylation observed during oral malignant transformation represents a departure from the typical methylation patterns observed in healthy cells. However, cancer cells seem capable of maintaining unique methylation homeostasis specific to their malignant phenotype, which could contribute to their unchecked growth. Targeting this cancer-specific methylation homeostasis may represent a potential intervention to alter the proliferation of cancer cells.

### DNMT1 knockdown remodels a highly disrupted and sheer genome-wide DNA hypomethylation pattern in OSCC

**Fig. 4:**
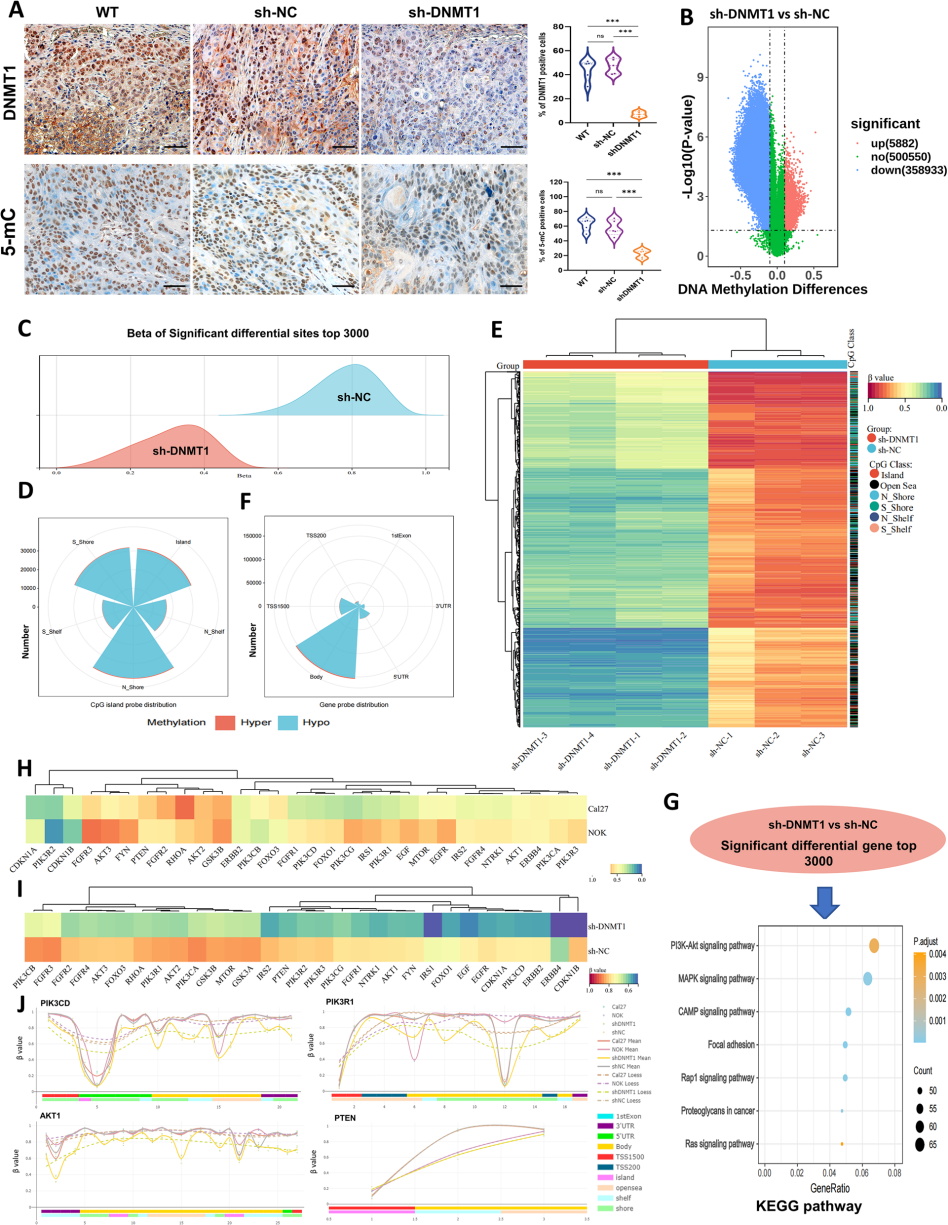
DNMT1 targeting remodeled an extensive and sheer genome-wide DNA hypomethylation pattern in OSCC. **A** Representative IHC images and analysis of DNMT1 and 5-mC in xenografted OSCC tumors. *n* = 5 mice in each group. Scale bars, 50 μm. **B** Volcano plots from DNA methylation 850 k chip showing significantly differential DNA methylation sites in sh-DNMT1 cells compared to sh-NC Cal27 cells. **C** Ridge plot showing the β value distribution of the top 3000 significantly differential sites in sh-DNMT1 and sh-NC Cal27 cells. **D** Nightingale rose chart showing the distribution of all DMSs with CpG island probes. **E** Heatmap showing the top 1000 differential CpG sites with the absolute differences in β values. The class of CpGs (in relation to CpG islands) is shown on the right of the heatmap. **F** Nightingale rose chart showing the proportion of all significant DMSs with gene probe distribution. **G** Bubble chart showing KEGG enrichment of DEGs related to DNA methylation sites. The differentially methylated sites distributed on CpG islands were sorted by β value difference, and the top 3000 related genes were selected for enrichment analysis. **H** and **I** Heatmap showing the mean methylation levels at differentially methylated sites of genes in the PI3K-AKT pathway between Cal27 and NOK cells, and between sh-DNMT1 and sh-NC cancer cells. **J** All significantly DMSs were enriched in the genes *PIK3CD*, *PIK3R1*, *AKT1* and *PTEN*, which are representative key genes in the PI3K-AKT pathway. Solid lines, the mean β values of each cell line; dotted lines, the β values loess of each cell lines. *P < 0.05, **P < 0.01, and ***P < 0.001 by unpaired Student’s* t* test or one-way ANOVA with Tukey’s multiple comparison test

As described above, OSCC tissues and cell lines exhibit DNMT1 overexpression alongside global DNA hypomethylation. Furthermore, silencing DNMT1 effectively inhibited tumor growth. Subsequent 5-mC detection in the shrunken OSCC tumors derived from DNMT1 knockdown mice revealed a substantial decrease in 5-mC expression (Fig. 4A). This finding suggested that the hypomethylated status, contingent on DNMT1, is associated with tumor suppression.

To further figure out how DNMT1 remodeled global DNA hypomethylation in OSCC, we compared sh-DNMT1 cancer cells to sh-NC cells using a DNA methylation 850 k chip. Strikingly, sh-DNMT1 cancer cells exhibited extensive and nearly complete genome-wide DNA hypomethylation. This was evident from the identification of 358,933 differentially hypomethylated sites, accounting for approximately 61.02 times of differentially hypermethylated sites in comparison to sh-NC cancer cells (Fig. 4B). The distribution of β values for the top 3000 significant DMSs resulted in the near-complete elimination of DNA methylation in OSCC cells (Fig. 4C). These findings provide further evidence that DNMT1 is an essential enzyme that regulates DNA methylation; any alteration or interruption in DNMT1 activity is strongly associated with changes in DNA methylation [14, 42]. However, the significant DNA hypomethylation observed in cancer cells following DNMT1 knockdown seems to contradict the prevailing notion that DNA hypomethylation may be linked to cancer initiation [43]. Actually, when revisiting the previously mentioned correlation analysis between DNA methylation and patient survival, it was observed that either a substantial decrease or increase in overall DNA methylation leads to an improved survival rate in the context of global DNA hypomethylation among OSCC patients. Our observation somewhat aligns with the hypothesis that extensive alterations in the DNMT1-mediated DNA hypomethylation pattern are linked to a tumor-suppressive effect.

To ascertain the potential underlying cause in greater detail, we proceeded to deduce the blueprint of the global DNA hypomethylation pattern of DNMT1 remodeling. The majority of differential hypomethylation sites caused by DNMT1 targeting were located at CpG island shores rather than CpG islands (Fig. 4D; Fig. S 5A). The observed differences in the distributions of the primary probes (Fig. S 3C) and the DMSs between OSCC cells and NOK cells were notable (Fig. S 5B). When the top 1000 DMSs were assessed, sh-DNMT1 cancer cells exhibited complete DNA hypomethylation, and a few DMSs were distributed at CpG islands but at island shores (Fig. 4E). Analysis of the distribution of functional gene regions revealed that DMSs were predominantly situated within the gene body (Fig. 4F; Fig. S 5A), whereas the top 3000 DMSs were primarily found within the gene body, followed by the transcription start site 1500 (TSS1500) region (Fig. S 5B). Compared to NOK cells, sh-DNMT1 cancer cells also exhibited more pronounced hypomethylation than sh-NC cells; the majority of these differential hypomethylation sites were at CpG island shores and gene bodies (Fig. S 5B).

Based on these results presented, it can be concluded that the downregulation of DNMT1 widely disrupted the homeostasis of global DNA methylation and led to a direct and persistent decrease in OSCC cells; this hypomethylation was characterized by a highly dispersed pattern of distribution transformation. Due to these extraordinary alterations, cancer cells may struggle to maintain proliferation. KEGG enrichment analysis and GO biological process analysis provided additional support for this finding. Both sets of DMGs were associated with various biological processes, including cell population proliferation, apoptotic processes, and specific signal transduction, with PI3K-AKT being the most prominent (Fig. 4G; Fig. S 5C). We then examined the methylation alterations in the PI3K-AKT pathway, and found that while the associated DMSs were slightly hypomethylated in cancer cells compared with those in NOK cells (Fig. 4H), DNMT1 knockdown resulted in extensive hypomethylation of critical genes (Fig. 4I). Key genes implicated in the activation of the PI3K-AKT signaling pathway, such as *PIK3CD*, *PIK3R1* and *AKT1*, showed dynamic hypomethylation; conversely, the inhibitory gene *PTEN* exhibited a pattern consistent with that observed in NOK cells (Fig. 4J; Fig. S 6A). Collectively, the aforementioned findings suggest that DNMT1 knockdown can directly remodel the global DNA hypomethylation in OSCC cells, leading to a pervasive disorder but sheer genome-wide DNA hypomethylation pattern subsequent to PI3K-AKT signaling alteration.

### DNMT1-remodeled global DNA hypomethylation mediates PI3K-AKT inhibition and enhances tumor growth suppression

**Fig. 5:**
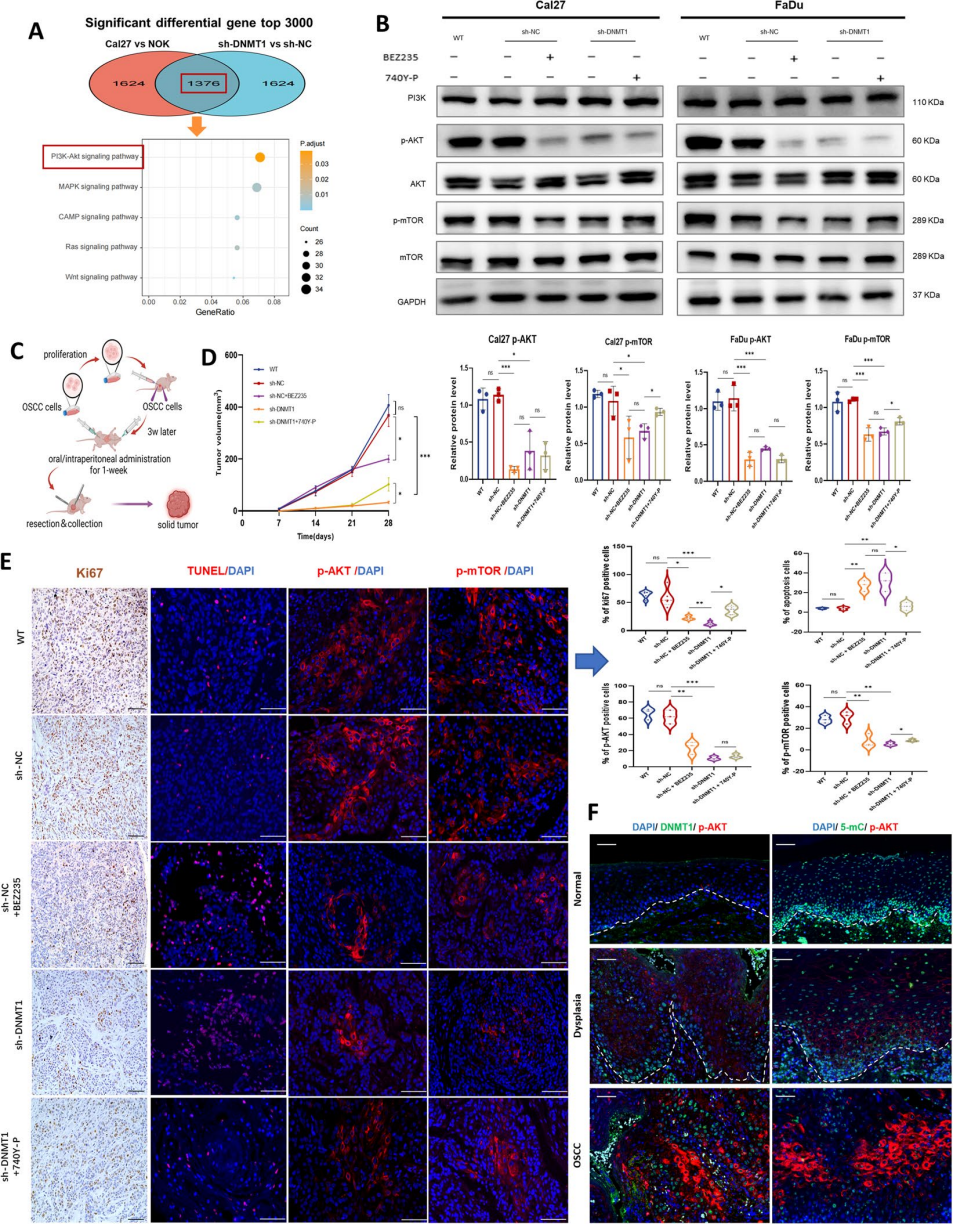
The PI3K-AKT pathway is involved in DNMT1-remodeled DNA hypomethylation pattern to regulate oral neoplastic transformation and tumor growth. **A** Bubble chart showing KEGG enrichment of the top 3000 overlapping genes related to differential DNA methylation sites between Cal27 and NOK and between sh-DNMT1 and sh-NC cells. **B** Western blot analysis of the indicated proteins in Cal27 and FaDu cells under the different conditions shown in the graph. Cells were treated with 1 µM BEZ235 or 25 µg/ml 740 Y-P for 24 h. GAPDH was used as an internal control. Upper panel: representative blots; lower panel: The densitometry quantification of the means ± SDs of three independent experiments. **C** Schematic showing the xenografted OSCC model. *n* = 5 mice in the WT, sh-NC, sh-DNMT1 and sh-DNMT1 + 740 Y-P groups, and *n* = 3 mice in the sh-NC + BEZ235 group. **D** Tumor growth curve. **E** Representative staining images and quantification of Ki67, TUNEL, p-AKT and p-mTOR staining in xenografted OSCC tumors. Scale bars, 100 μm. **F** Representative mIHC images of three channels, namely, DAPI, DNMT1 or 5-mC, and p-AKT in oral human samples including normal, dysplastic and OSCC tissues. Scale bars, 50 μm. *P < 0.05, **P < 0.01, and ***P < 0.001 by one-way ANOVA with Tukey’s multiple comparison test

Through KEGG classification analysis, we identified the PI3K-AKT signaling pathway as an essential pathway associated with the DNMT1-specific DNA methylation pattern. This finding was further supported by the overlap of the top 3000 DMGs between Cal27 cells and NOK cells, and between sh-DNMT1 cells and sh-NC Cal27 cells (Fig. 5A; Fig. S5D). Considering the DMSs associated with the PI3K-AKT signaling pathway, all of these DMSs were hypomethylated following DNMT1 knockdown, and the majority were located at CpG island shores and within gene bodies, especially AKT-related DMSs (Fig. S 6B). This finding suggested that the activation of PI3K-AKT may be inhibited, as the methylation levels within gene bodies and methylations specific to tumors that occur at CpG island shores are often positively correlated with gene expression [44, 45].

The expression levels of total and phosphorylated proteins were then assessed, revealing a significant reduction in phosphorylated AKT and mTOR in sh-DNMT1 cancer cells, although the overall protein expression levels of PI3K and AKT did not exhibit substantial alterations (Fig. 5B). This implies that the activation of PI3K-AKT is significantly hindered due to DNMT1-remodeled DNA hypomethylation. A rescue experiment was performed by using a PI3K agonist (740 Y-P) in sh-DNMT1 cells. These results indicated that 740 Y-P was able to partially restore the phosphorylation levels of mTOR in sh-DNMT1 cells (Fig. 5B). This finding provided evidence that DNMT1 regulates PI3K-AKT signal transduction and suggested the possible involvement of other mechanisms modulated by DNMT1.

PI3K inhibitors have been verified to restrain tumor growth in several SCCs [46, 47]. Then, we performed in vivo investigations utilizing a xenografted OSCC mouse model in which PI3K was inhibited with BEZ235 (Fig. 5C). A reduction in tumor growth was observed after both DNMT1 and PI3K inhibition (Fig. 5D). Intriguingly, the suppressive effect on tumor shrinkage was more prominent in cases of DNMT1 knockdown compared to PI3K inhibitor treatment. When PI3K agonists were applied to DNMT1-silenced mice, only a modest increase in tumor growth was observed (Fig. 5D). The observed reduction in the number of proliferative cells and increase in the number of apoptotic cells within the sh-DNMT1 group provided evidence of a significant tumor-suppressing effect (Fig. 5E). Moreover, DNMT1 knockdown effectively blocked the PI3K-AKT signaling pathway in subcutaneous tumors, as indicated by the reduced phosphorylation of AKT and mTOR (p-AKT and p-mTOR). PI3K-AKT signal transduction was partially restored upon treatment with PI3K agonists, but was suppressed compared to that in the sh-NC group (Fig. 5E). This discovery provides additional evidence for the existence of other mechanisms that inhibit tumor growth as a result of DNMT1-specific DNA hypomethylation. Additionally, we examined the colocalization of DNMT1 or 5-mC and p-AKT in human samples, encompassing normal, dysplastic and OSCC tissues. During oral carcinogenesis, an increase in p-AKT was observed, followed by the overexpression of DNMT1 and the loss of 5-mC expression (Fig. 5F).

### DNMT1 targeting results in greater tumor suppression through the additional inhibition of the CDK2-Rb pathway

To determine the additional molecular mechanisms that underlie DNMT1 inhibition and resulting alterations in DNA methylation, we conducted an intersectional meta-analysis using the TCGA database and 4 GEO datasets, and then overlapped with the DEGs linked to DNMT1 from our 850 k chip profile. This analysis identified a total of 152 DEGs connected to both DNMT1 and global DNA methylation (Fig. S 7A). These DEGs were subsequently validated to be associated with DNA replication, cell cycle, base excision repair and apoptosis pathways by KEGG enrichment analysis (Fig. S 7B). By employing PPI analysis and implementing a network filter, we identified a set of five distinct node molecules, namely, CDK2, GSK3B, ABL1, NFKB1, and CREBBP (Fig. S 7C). Only the mRNA expression of CDK2 and GSK3B was significantly greater in the OSCC samples from the TCGA database, as compared to the normal tissues (Fig. S 7D). Both exhibited a positive correlation with DNMT1 expression in OSCC (Fig. S 7E).

**Fig. 6:**
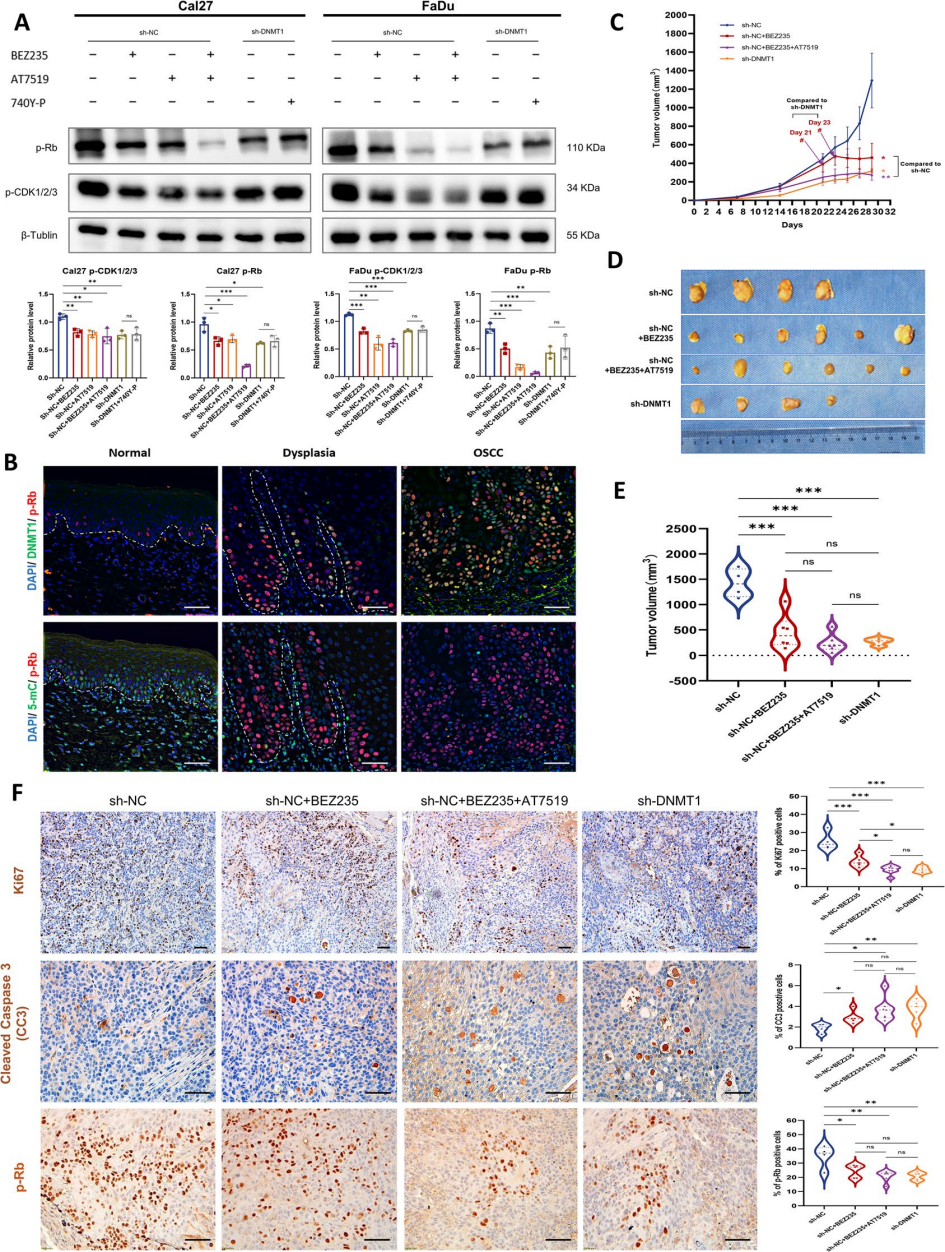
Restraining the CDK2-Rb signaling pathway contributed to the enhanced tumor-suppression caused by DNMT1-remodeled global DNA hypomethylation.** A** Western blot analysis of the indicated proteins in Cal27 and FaDu cells under different conditions is shown in the graph. Cells were treated with 1 µM BEZ235 or 25 µg/ml 740 Y-P for 24 h or with 1 µM AT7519 for 8 h. β-Tubulin was used as an internal control. Upper panel:: representative blots; lower panel: The densitometry quantification of the means ± SDs of three independent experiments. **B** Representative mIHC images of three channels, namely, DAPI, DNMT1 or 5-mC and p-Rb in oral human samples including normal, dysplastic and OSCC tissues. Scale bars, 50 μm. **C** Tumor growth curve. *n* = 5 mice for the sh-NC + BEZ235 and sh-NC BEZ235 + 740 Y-P groups, and *n* = 4 mice for the sh-NC and sh-DNMT1 groups. #*P* < 0.05 by unpaired Student’s t test. **D** and** E** Presentation of gross xenograft tumors (d) and tumor volume statistics (e) at the endpoint of the study. **F** Representative IHC images and analysis of Ki67, cleaved caspase 3 (CC3) and p-Rb in xenograft OSCC tumors. Scale bars, 50 μm. *P < 0.05, **P < 0.01, and ***P < 0.001 by one-way ANOVA with Tukey’s multiple comparison test

CDK2 plays a critical role in cancer progression by regulating the cell cycle and phosphorylating retinoblastoma protein (Rb), particularly during the G1–S phase transition [48]. A reduction in the phosphorylation levels of CDK1/2/3 and Rb was observed in sh-DNMT1 cancer cells compared to sh-NC cells (Fig. 6A), confirming the additional inhibition of CDK2-Rb signal transduction. Furthermore, a PI3K inhibitor was shown to function in the deactivation of CDK2-Rb, and the combined use of PI3K and CDK2 inhibitors (BEZ235 and AT7519) had the most pronounced inhibitory effect, indicating a potential interplay between PI3K-AKT and CDK2-Rb signaling pathways [49]. However, the PI3K agonist did not fully restore the activation of CDK2-Rb, implying that DNMT1-mediated repression of CDK2-Rb and PI3K-AKT may occur in parallel. Also, we observed a consistent increase in the p-Rb level during neoplastic transformation, along with elevated DNMT1 expression and attenuated 5-mC (Fig. 6B), confirming the regulatory effect of the DNMT1-DNA methylation pattern on CDK2-Rb pathway.

We further conducted concurrent treatment in xenografted mice with BEZ235 and AT7519, targeting the dual suppression of the PI3K-AKT and CDK2-Rb pathways, as a positive control. Mice treated with combination inhibitors exhibited comparable suppression of tumor growth to mice bearing sh-DNMT1 tumors. Compared to mice that received a single PI3K inhibitor, mice in which DNMT1 was silenced also exhibited the slowest tumorigenesis and superior control of tumor growth in the earlier intervention (Fig. 6C). At the endpoint of the study, all three groups of tumor-bearing mice subjected to the intervention exhibited significant shrinkage in tumor volume compared to those bearing sh-NC tumors. However, the sh-DNMT1 group did not exhibit superior tumor suppression compared to the other two groups treated with inhibitors (Fig. 6, D and E). This lack of superiority may be attributed to the reversible nature of DNMT1, which could counteract the effects of DNMT1 gene silencing. Nevertheless, we did observe a decrease in cell proliferation and an increase in cell apoptosis among these three experimental groups, providing compelling evidence for the anticancer effectiveness of these interventions. The proportion of Ki67-positive cells in sh-DNMT1 tumor-bearing mice was found to be mitigated, offering further evidence for the enhanced anticancer efficacy of DNMT1 silencing (Fig. 6F). Moreover, the phosphorylation of the Rb protein decreased in the sh-DNMT1 group (Fig. 6F), further supporting our discovery that DNMT1-remodeled DNA hypomethylation can inhibit tumor growth by concurrently suppressing the PI3K-AKT and CDK2-Rb signaling pathways.

### DNMT1 knockdown further induces GSK3β inactivation to facilitate cell apoptosis and to antagonize PI3K inhibition-induced insulin feedback

**Fig. 7:**
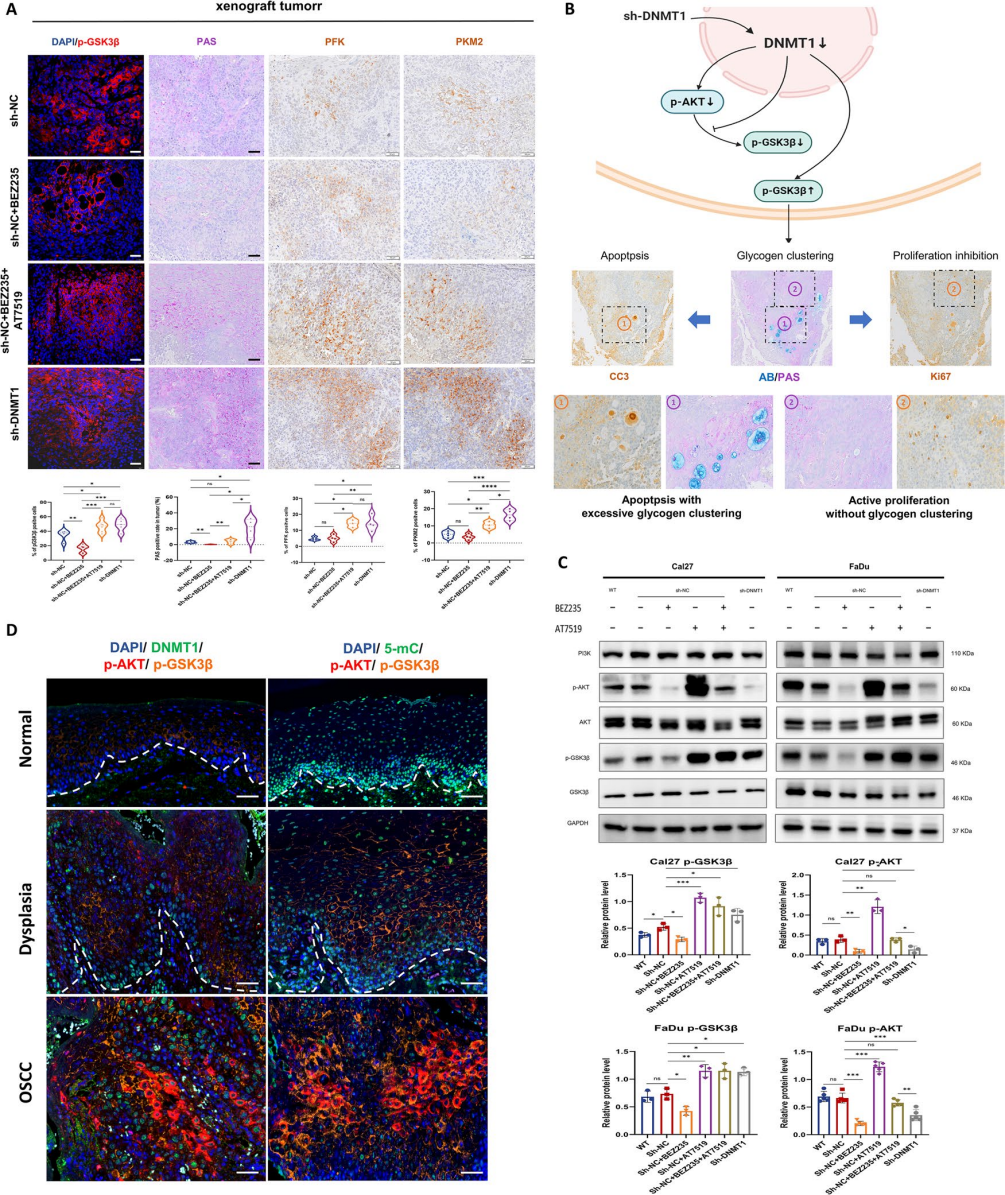
The GSK3β inactivation by DNMT1 knockdown leads to excessive glycogen clustering and apoptosis in tumors.** A** Representative IF images for p-GSK3β and PAS/IHC images for glycogen, PFK and PKM2 in xenografted OSCC tumors. Below are the corresponding statistical analysis results. Scale bars, 50 μm. **B** As shown in the schematic, DNMT1 knockdown prevents GSK3β activation downstream of PI3K inhibition by upregulating p-GSK3β, leading to excessive glycogen clustering in the tumor (AB-PAS staining), which is located around apoptosis and far from proliferation (IHC staining). **C** Western blot analysis of the indicated proteins in Cal27 and FaDu cells under the different conditions shown in the graph. Cells were treated with 1 µM BEZ235 for 24 h and 1 µM AT7519 for 8 h. GAPDH was used as an internal control. Upper panel: representative blots; lower panel: densitometry quantification of the means ± SDs of at least three independent experiments. **D** Representative mIHC images showing DAPI, DNMT1 or 5-mC, p-AKT and p-GSK3β in oral human samples, including normal, dysplastic and OSCC tissues. Scale bars, 50 μm. *P < 0.05, **P < 0.01, and ***P < 0.001 by one-way ANOVA with Tukey’s multiple comparison test

Regarding GSK3B, the other gene candidate strongly associated with DNMT1-mediated DNA hypomethylation, our observations revealed significant changes in GSK3β phosphorylation at Ser9 (p-GSK3β) in xenografted tumors. When either DNMT1 was knocked down or a combination of PI3K and CDK2 inhibitors was applied, there was a notable increase in p-GSK3β. Conversely, PI3K inhibition resulted in a noticeable decrease in p-GSK3β (Fig. 7A). GSK3β serves as an important regulatory enzyme in maintaining cell metabolic balance, especially in processes such as glycogen synthesis and glycolysis in cancer cells [50, 51]. Its inactivation occurs through the phosphorylation of Ser9, facilitating glucose synthesis [52]. We subsequently examined increased glycogen clustering in sh-DNMT1 tumor tissues, similar to those treated with combined inhibitors, but notably more than in tumors treated in solely with PI3K inhibitors or vehicle. Besides, PI3K inhibitor-treated tumors exhibited very little glycogen accumulation (Fig. 7A). Both DNMT1 knockdown and combined PI3K and CDK inhibition led to increased levels of glycolysis in tumor tissues, as indicated by increased numbers of PFK- and PKM2- positive cells. These cells were highly located in regions with large glycogen deposits (Fig. 7, A and B). Interestingly, these cells showing glycogen clustering and heightened glycolysis were predominantly found near the apoptotic tumor area and far from the proliferative region (Fig. 7B). Taken together, these results implied that the DNMT1-induced GSK3β inactivation promotes cancer cell death through abnormal glycogen metabolism.

While GSK3β is typically regulated by AKT signaling [53], our in vitro experiments showed that both targeting DNMT1 and combined inhibition of PI3K and CDK2 reversed the increase in p-GSK3β expression in OSCC cells when compared to that resulting from single PI3K inhibition. Interestingly, only DNMT1 knockdown continuously suppressed PI3K-AKT activation (Fig. 7C). In contrast, a single CDK2 inhibitor had a stimulating effect on AKT phosphorylation, and combined CDK2 inhibitors showed no effect on PI3K-AKT activation. These findings additionally imply that DNMT1 silencing could restore the signaling equilibrium by simultaneously inactivating PI3K-AKT, CDK2-Rb and GSK3β. Moreover, in human oral multicarcinogenesis samples, we observed the extensive changes in the expression of p-GSK3β along with changes in p-AKT, and DNMT1 expression and DNA methylation changing (Fig. 7D). This finding provides further evidence supporting the involvement of the internal PI3K-AKT-GSK3β signaling pathway of in OSCC.

**Fig. 8:**
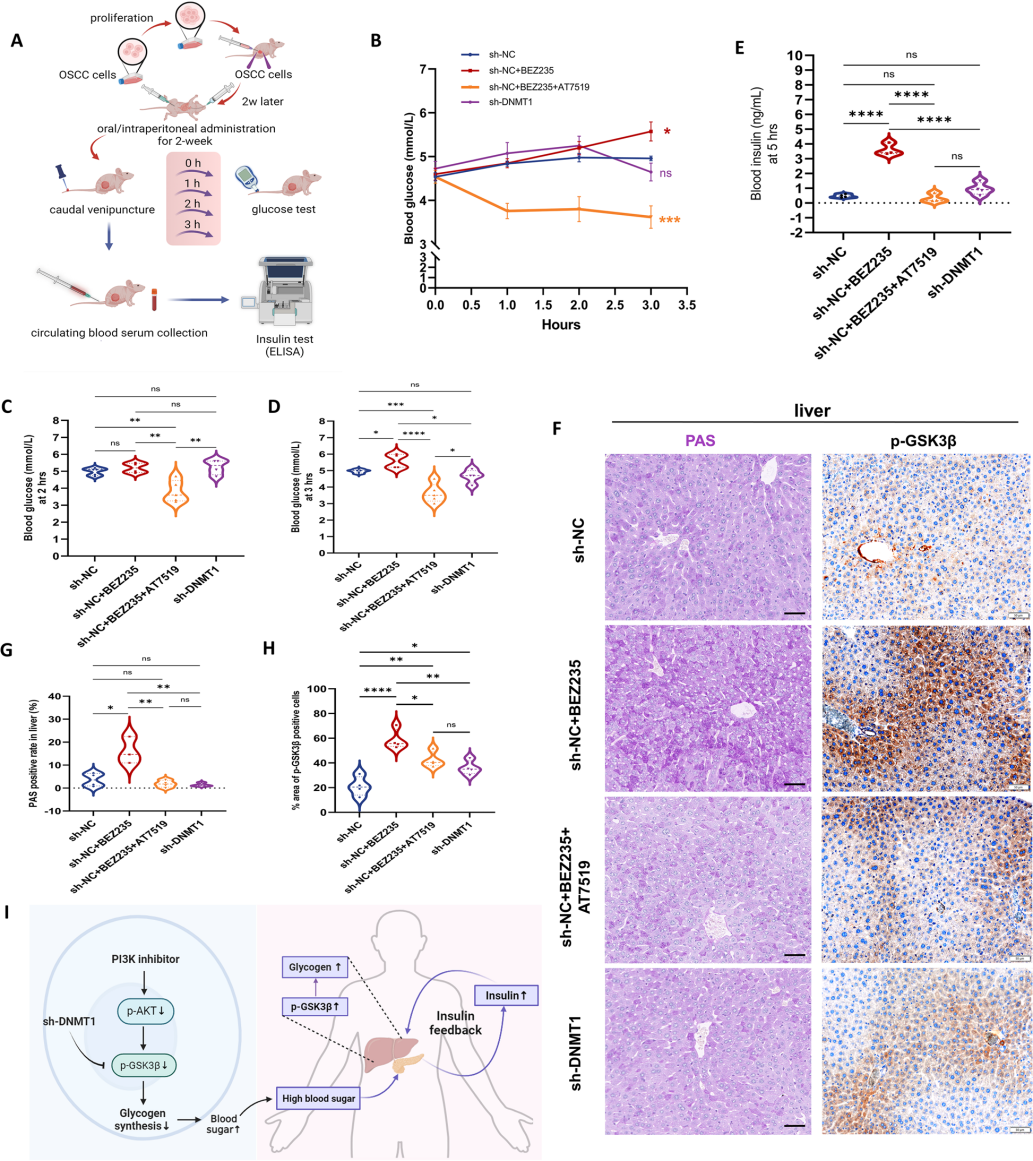
The GSK3β inactivation by DNMT1 knockdown antagonizes the insulin feedback resulting from PI3K inhibition. **A** Experimental schematic of blood glucose and serum insulin detection in tumor-bearing mice. *n* = 4 mice for each group. **B** Line chart of blood glucose within 3 h after administration. **C** and** D** Blood glucose of mice at 2 h (C) and 3 h (D) after administration respectively. **e** Serum insulin levels of the mice at 5 h after administration. **F–H** Representative PAS/IHC images (f) and analysis (g, h) of glycogen/p-GSK3β in mouse livers 5 h after administration. Scale bars, 50 μm. **I** As shown in the schematic, a PI3K inhibitor activates GSK3β through dephosphorylation, hindering glycogen synthesis and causing detrimental hyperglycemic effects. Elevated blood glucose levels can induce insulin feedback, culminating in hepatic glycogen accumulation. DNMT1 silencing can block this process by promoting GSK3β phosphorylation. *P < 0.05, **P < 0.01, and ***P < 0.001 by a one-way ANOVA with Tukey’s multiple comparison test

Systemic glucose disruption secondary to PI3K inhibitors has been evident from their use in animal cancer models and clinical trials. This disruption can limit the effectiveness of anticancer therapies [54, 55]. In our OSCC mouse model, we carried out experiments to observe alterations in blood glucose levels (Fig. 8A). The administration of a PI3K inhibitor gradually induced hyperglycemia. Conversely, when PI3K inhibitors were used in combination with CDK2 inhibitors, there was a rapid and notable decrease in blood glucose levels (Fig. 8, B-D). At the 5-h time point after treatment, the serum insulin level in mice treated with BEZ235 remained consistently elevated, while a dramatic fall was observed in mice treated with combined inhibitors (Fig. 8E). Moreover, tumor-bearing mice in which DNMT1 was targeted exhibited a notable stability in both blood glucose and serum insulin levels.

As evidenced in prior research, the transient hyperglycemia caused by PI3K inhibition often remains within a few hours, as insulin feedback mechanisms kick in to restore normal glucose homeostasis [54]. Thus, we examined the glycogen synthesis conditions in the liver and found that only tumor-bearing mice treated with a PI3K inhibitor exhibited significantly high glycogen levels (Fig. 8, F and G), accompanied by elevated expression of p-GSK3β (Fig. 8, F and H). Conversely, mice experiencing hypoglycemia due to combined inhibitor treatment exhibited reduced liver glycogen storage and lower levels of p-GSK3β, further suggesting that liver glycogenolysis contributed to the recovery of normal blood sugar. However, sh-DNMT1 tumor-bearing mice exhibited a slightly greater level of p-GSK3β than nontreated xenografted mice, suggesting that glycogen depredation in sh-DNMT1 tumors themselves may also trigger compensatory liver glycogen metabolism to a certain extent. Together, these findings suggest that a suppressive intervention targeting DNMT1 is most likely to reduce the adverse toxicity to maintain normal glucose balance in the context of PI3K inhibition-induced hyperglycemia, further enhancing the effectiveness of anticancer treatments.

### The DNMT1-mediated signaling synergia pattern and mechanism schematic in oral carcinogenesis and anti-cancer efficacy

Based on the results above, it appears that there are synergetic signal transduction pathways involving PI3K-AKT, CDK2-Rb, and GSK3β during oral neoplastic transformation and in treating OSCC, influenced by the DNMT1-DNA methylation pattern. OSCC, a heterogeneous solid tumor, progresses through multiple steps of carcinogenesis from normal to precancerous to cancerous lesions. To further validate this synergistic signaling synergy pattern in oral malignant transformation, we performed the single-cell transcriptome analysis using the GSE181919 dataset, to better understand the genetic variation of tumor heterogeneity at the single-cell level.

**Fig. 9:**
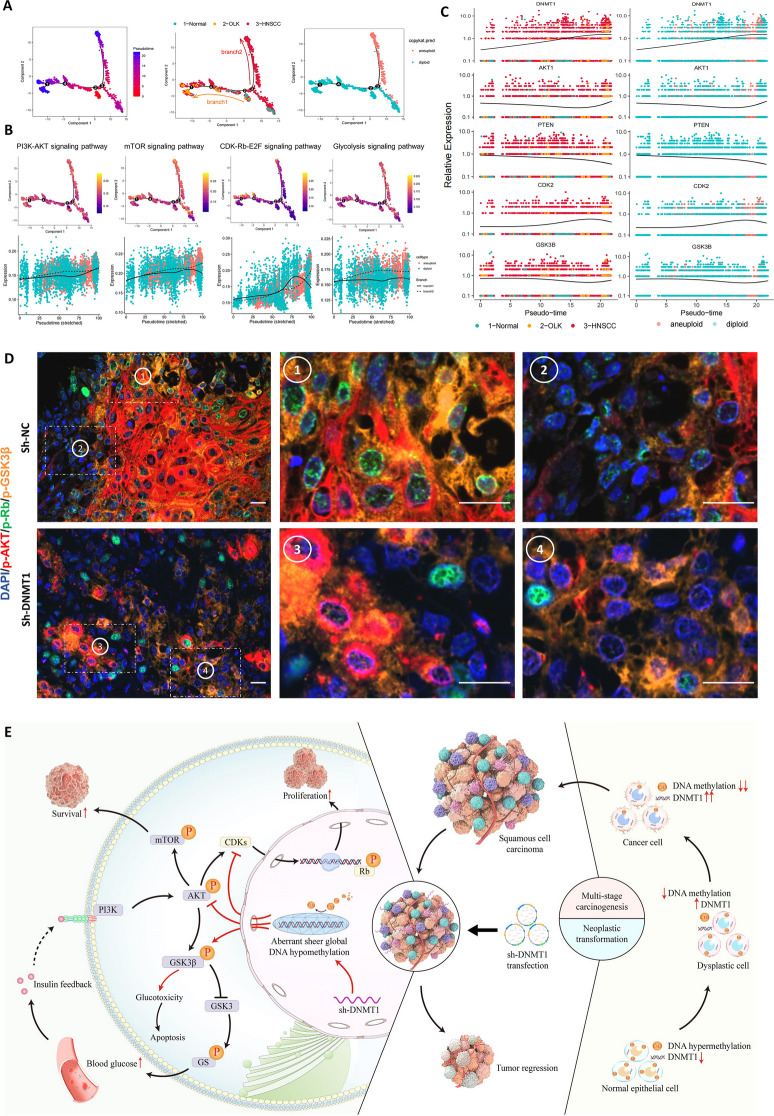
The DNMT1-mediated signaling synergy pattern and schematic in oral carcinogenesis and anticancer efficacy. **A** Pseudotime trajectory of epithelial cells, with each color coded for pseudotime (left), groups (middle), and copykat.pred (right) **B** Pseudotime trajectory analysis of the AUCell score of PI3K-AKT, mTOR, CDK-Rb-E2F and glycolysis signaling pathways. **C** Pseudotime trajectory analysis of the expression of DNMT1, PTEN, AKT1, CDK2 and GSK3B. **D** Representative mIHC staining images of DAPI, p-AKT, p-Rb and p-GSK3β in xenografted OSCC tumors. Scale bars, 20 μm. **E** Schematic indicating that the DNMT1- dependent global DNA methylation pattern functions in oral carcinogenesis and treatment of OSCC, as well as the collaborative signal transduction involved. Red arrows represent the effects of sh-DNMT1; black arrows represent natural biological processes. This schematic image was created by BioRender

We first separated epithelial cells from normal, OLK and HNSCC tissues (Fig. S 8A-E), respectively. Considering the presence of cell heterogeneity in the epithelial cell population, we reclassified diploid and aneuploid cells (Fig. S 8F) since the latter can completely represent the malignant populations. Pseudotime trajectory analysis revealed two progressive branches from normal epithelial cells, one leading to a precancerous state and the other to a cancerous state, with aneuploid cells prominently located in the latter (Fig. 9A; Fig. S 8, G-I). Collaborative signaling pathways, including PI3K-AKT, mTOR, CDK-Rb-E2F and Glycolysis were activated as the epithelial cells progressed toward a cancerous state (Fig. 9B, Top). Comparing the trend towards the precancerous state, all involved signal transduction pathways involved in the malignant process showed persistent activation (Fig. 9B, bottom). Furthermore, pseudotime trajectory analysis of individual key genes regulating this signaling synergia pattern confirmed increased expression of DNMT1, AKT1, CDK2 and GSK3B during epithelial carcinogenesis, while the expression of the negative regulatory gene PTEN decreased (Fig. 9D). Thus, the results from single-cell transcriptome analysis further confirmed the critical role of signal synergistic function pattern in oral carcinogenesis.

In the xenografted tumors, we also utilized multiplex immunohistochemical technique to determine the colocalization of the core regulatory markers at the protein functional level (Fig. 9D). In actively proliferative OSCC tumors, cancer cells exhibit high levels of p-AKT, p-Rb, and p-GSK3β. Most cancer cells showed frequent colocalization of these three markers, regardless of whether they were either highly or weakly expressed (Fig. 9D, top). Upon DNMT1 targeting, the xenografted tumors bared smaller cancerous lesions scattered, with reduced expression of p-AKT and p-Rb but still extensive expression of p-GSK3β. In particular, the alterations in both p-Rb and p-GSK3β were independent of the changes in AKT phosphorylation (Fig. 9D, bottom). These findings reaffirmed the potent regulatory role of DNMT1 in coordinating the multiple signals transduction.

After compiling the aforementioned findings, we present a schematic elucidating the role of DNMT1 in oral malignant transformation and in controlling tumor growth. As illustrated (Fig. 9E), DNMT1 expression gradually rises increases in tandem with genome-wide DNA hypomethylation, which triggers the multistage carcinogenesis of OSCC. Targeting DNMT1 leads to the formation of an aberrant and widespread DNA hypomethylation state, inducing a specific signaling synergia. These pathways involve the dual inhibition of PI3K-AKT and CDK2-Rb, along with the inactivation of GSK3β, which regulates reduced cell proliferation and increased cell apoptosis, thus impeding tumor growth. Additionally, targeting DNMT1 potentially counteracts the pharmacological toxicity of hyperglycemia and insulin feedback stemming from PI3K inhibition. This process is activated by inducing supererogatory GSK3β inactivation, leading to sustained blockade of PI3K-AKT activation. All these processes represent a signaling synergy orchestrated by DNMT1, acting as a gatekeeper to effectively restrain tumor growth through enhancing efficiency and reducing toxicity.

## Discussion

Oral squamous cell carcinoma (OSCC) represents a highly heterogeneous and aggressive cancer type that undergoes a multistage neoplastic transformation process. This process encompasses genomic instability and epigenetic aberrations, leading to intricate alterations in gene expression, anomalies in signaling pathways, and changes in physiological functions [2, 56]. Global DNA hypomethylation, a prominent feature of malignancies, is largely dependent on the stable maintenance by DNMT1 [17, 57, 58]. Building upon our precious prediction of DNMT1 as a potential marker of OSCC progression, this present study proposed that DNMT1 regulates oral carcinogenesis and OSCC growth through a novel mechanism involving the remodeling of specific global DNA methylation patterns to initiate multiple signaling collaborations. Our findings revealed that targeting DNMT1 in oral cancer cells resulted in near-complete genome-wide DNA hypomethylation, triggering the dual attenuation of PI3K-AKT and CDK2-Rb and collaborative phosphorylation of GSK3β. This cascade eventually produces a remarkable anticancer effect-enhancing and toxicity-reducing.

DNMT1 is commonly overexpressed in various cancers and is often linked to poor patient prognosis, as indicated by previous studies [59–62]. In our study, we have demonstrated a new discovery regarding DNMT1 expression, which progressively increased throughout epithelial multistage carcinogenesis. While OSCC tissues exhibited significantly increased DNMT1 expression, we observed a dynamic correlation between DNMT1 expression, mortality risk, and survival hazard ratio, taking into account complex clinical variables. Notably, relatively lower DNMT1 expression was associated with a more favorable prognosis in OSCC individuals. When DNMT1 was silenced or targeted with inhibitors, OSCC cells displayed quite hysteretic tumorigenic capacity and restricted tumor growth. These results provide compelling evidence that DNMT1 represents a potent target for controlling OSCC progression.

Host cells possess a specific mechanism known as global DNA methylation homeostasis to maintain the stability of their cellular genome, a process heavily dependent on the accurate maintenance carried out by DNMT1 [63]. In cancer cells, the phenomenon of genome-wide hypomethylation generally persists throughout biological processes but has a limited efficacy in maintenance, leading to a selective and imbalanced DNA methylation status during cell division [9, 63]. Our findings confirmed that the progression of oral epithelial carcinogenesis is accompanied by a gradual decrease in DNA methylation, ultimately resulting in the genome-wide hypomethylation pattern observed in OSCC. Additionally, we investigated a undulatory link between global DNA hypomethylation and OSCC prognosis. A significant alteration in overall DNA methylation, either a substantial decrease or increase, may correlate with an improved survival rate. Unexpectedly, we observed a nearly complete hypomethylation pattern, remodeled by DNMT1 silencing, alongside effective suppression of cancer. This observation underscores the crucial role of DNMT1 in preserving the preexisting methylation pattern [15, 63] and highlights that its inactivation can directly trigger dysregulation of global DNA methylation in OSCC. We also observed that the genome-wide methylation remodeling pattern specific to DNMT1 may not follow a restorative trajectory towards normal cells. Instead, it has the potential to significantly disrupt DNA methylation homeostasis during DNA replication, ultimately impeding the proliferation of cancer cells or inducing cell death. Besides, the coexistence of DNMT1 overexpression and genome-wide hypomethylation in oral cancer cells may reflect that, given the low maintenance efficacy of DNA methylation [63], the upregulation of DNMT1 functions as a self-compensatory mechanism to maintain DNA methylation equilibrium.

In addition to a sheer decline in genome-wide DNA methylation level, we observed a striking expansion in the distribution of hypomethylated CpG sites, particularly at CpG island shores and within gene body regions. These regions are crucial functional components involved in gene expression regulation [44]. Some scholars have noted that most tissue-specific or cancer-associated DNA methylation changes tend to occur at CpG island shores [64], although the precise mechanism underlying these changes has yet to be fully elucidated. On the other hand, the disruption of global DNA methylation caused by DNMT1 alteration triggered a series of biological processes and changes in signal transduction, which were sufficient to change the malignant behavior of OSCC cells. In this scenario, the PI3K-AKT signaling pathway is strongly activated. Our study confirmed the promotion of its activation in conjunction with epithelial carcinogenesis, as well as its inhibition following DNMT1 knockdown, resulting in decreased phosphorylation of AKT and mTOR. This finding aligns with reports suggesting that DNMT1 overexpression could activate the PI3K-AKT signaling pathway to promote melanoma development [65, 66]. However, it's noteworthy that the total protein expression of PI3K, AKT and mTOR remained unaltered despite the reconstructed DNA methylation pattern. This observation is reminiscent of findings in *Arabidopsis*, where ectopic DNA methylation mediated by the bacterial SssI methyltransferase had little effect on transcription, despite the hypothesized link between modified DNA methylation at the gene body and gene expression [67].

The current study unveils an intriguing discovery indicating that DNMT1 knockdown more strongly inhibits tumor growth than does inhibition of the PI3K-AKT pathway alone. This finding implies that DNMT1 play a role in regulating other signaling pathways associated with oncogenes or cancer suppressor genes. CDK2, known for its crucial involvement in the cell cycle process, has emerged as a promising therapeutic target for treating cancer [68]. In this research, the concurrent suppression of the CDK2-Rb signaling pathway was confirmed following DNMT1 knockdown. This was evidenced by the reduction in phosphorylation levels of CDK1/2/3 and Rb proteins in sh-DNMT1 cancer cells. Activation of this pathway, indicated by increased p-Rb, was observed as tissues progressed from normal to dysplastic and OSCC tissues, highlighting its function in oral carcinogenesis. Furthermore, our research elucidated the tumor-promoting function of PI3K-AKT and CDK2-Rb activation, both of which can be suppressed by DNMT1-specific DNA hypomethylation. The interaction between PI3K-AKT and CDK2-Rb is indisputably advantageous for cancer cell growth. For instance, activated AKT can phosphorylate CDK inhibitors such as p21 [69] and p27 [70] to further activate Rb transcription, thus facilitating cell proliferation. In the context of DNMT1-mediated global DNA hypomethylation, our study put forth a proposal that the concurrent inhibition of these two pathways is partially independent. This is supported by the finding that the administration of a PI3K agonist did not fully restore CDK2-Rb activation. The observation of nonoverlapping patterns for immunofluorescent p-AKT and p-Rb in DNMT1-knockdown tissues provides additional evidence supporting this perspective.

Moreover, in xenografted OSCC tumors, both dual inhibition of PI3K and CDK2, as well as DNMT1 knockdown, resulted in an extra elevation of p-GSK3β ser9, leading to the blockade of GSK3β activation and promoting more glycogen storage [51, 52]. While increased glycogen synthesis and glycogenolysis in cancer cells are typically associated with cancer progression [71], this study revealed unique findings regarding DNMT1-mediated glycogen deposition. Interestingly, DNMT1-mediated glycogen deposition was abnormally located in apoptotic cells around necrotic tumor areas, accompanied by enhanced glycolysis. These results demonstrate an exceptional mechanism by which glucotoxicity that promotes apoptosis, which is closely linked to DNMT1-induced GSK3β inactivation. Notably, under certain tumor-treating conditions, inhibited glycogen clustering has been shown to attenuate the anticancer effect [72]. Additionally, proliferating cancer cells generally do not exhibit PKM2-mediated glycolysis [73]. These findings provide some indirect support for the results obtained in this study but remain to be explored further.

PI3K inhibitors used in cancer treatment often induce hyperglycemia, triggering insulin feedback mechanisms that diminish their efficacy in treating cancer [54, 55]. GSK3β, a key regulator in the insulin receptor signaling pathway for the regulation of blood glucose levels, plays a crucial role in this process [74]. We observed that mice orally administered with a PI3K inhibitor exhibited elevated blood glucose levels accompanied by a corresponding increase in serum insulin levels as a feedback response. This finding is likely due to a biochemical mechanism whereby the inactivation of AKT inhibits the phosphorylation of GSK3β ser9 [75]. In the context of biological insulin feedback derived from hyperglycemia, liver glycogen synthesis increases through elevated phosphorylation of GSK3β, contributing to the recovery of normal glycemic homeostasis. Remarkably, we found that DNMT1 silencing led to a compensatory increase in p-GSK3β levels, as did the sustained suppression of PI3K-AKT activation. This combination may contribute to the maintenance of stable blood glucose levels. Conversely, additional inhibition of CDK2-Rb partially reactivated PI3K-AKT signaling, leading to an excessive reduction in blood glucose. These cumulative results suggest that DNMT1 has the capacity to concurrently and accurately modulate the signal transduction of PI3K-AKT, CDK2-Rb, and GSK3β- mediated glycogen metabolism. This mechanism contributes to the establishment of signaling synergia and an inherent balance governing cancer behavior, thereby improving anticancer effects and preventing adverse effects resulting from intercommunication between these pathways.

In summary, our study provides comprehensive data demonstrating that precise DNMT1-targeting disrupts global DNA methylation, forming as a vital approach to facilitate anticancer efficacy while minimizing potential toxic effects arising from signal crosstalk in targeted therapy. Mechanistically, we propose a functional model wherein DNMT1-remodeled genome-wide DNA hypomethylation patterns regulate oral malignant transformation and tumor growth, through signal collaborations involving PI3K-AKT, CDK2-Rb and GSK3β-mediated glycogen metabolism. Our findings suggest that targeted intervention against DNMT1 using compounds, biomaterials or nanomedicines holds promise as an alternative approach for OSCC therapy, regarding its pivotal role as a signal gatekeeper. Further investigations are warranted to discover how DNMT1 remodels specific DNA hypomethylation patterns, which will contribute to a deeper understanding of the underlying epigenetic mechanism driving OSCC progression. This research direction holds potential for identifying novel therapeutic targets and improving treatment outcomes in OSCC patients.

## Conclusions

In this study, we precisely simulated DNMT1-targeted interventions in cancer cells and validated their potent efficacy in suppressing tumor growth in an OSCC mouse model. Mechanistically, DNMT1 inhibition led to a reshaping of the genome-wide DNA hypomethylation pattern, which hindered the dual activation of PI3K-AKT and CDK2-Rb while inducing GSK3β inactivation. Compared to PI3K inhibitors, DNMT1 targeting demonstrated superior in *vivo* tumor suppression, mitigating the toxic effects of blood glucose variation caused by PI3K and PI3K-CDK inhibitor combinations. Analysis of human samples revealed a correlation between oral malignant transformation, elevated DNMT1 expression, and accumulating cancer-specific DNA hypomethylation. This was associated with collaborative signal transduction involving the PI3K-AKT, CDK2-Rb, and GSK3β pathways. DNMT1 targeting not only remodels the genome-wide DNA hypomethylation pattern, but also achieves enhanced anticancer efficacy and reduced toxicity by equilibrating signaling synergia. Our research highlights DNMT1 as a gatekeeper in determining OSCC destiny and treatment outcome, confirming its potential as a useful therapeutic target for OSCC. These findings contribute to a deeper understanding of the molecular mechanisms underlying OSCC progression and provide a basis for the development of more effective and targeted therapeutic strategies against this malignancy.

### Supplementary Information

**Supplementary Material 1.**

**Supplementary Material 2.**

## Data Availability

All the data needed to evaluate the conclusions in the article are presented in the article and/or the Supplementary Materials. The original data and materials used in the current study are available from the corresponding authors upon reasonable request.

## Abbreviations

OSCC: Oral squamous cell carcinoma
DNMT1: DNA methyltransferase 1
CC3: Cleaved Caspase-3
PFK: Phospho fructo kinase
IHC: Immunohistochemistry
IF: Immunofluorescence
TUNEL: Terminal deoxynucleotidyl transferase dUTP nick end labeling
PAS: Periodic acid-schiff
TCGA: The Cancer Genome Atlas
OLK: Oral leukoplakia
PCA: Principal component analysis
MDS: Multidimensional scaling
WGA: Whole-genome amplification
SWAN: Subset-quantile Within Array Normalization
DMSs: Differential DNA methylation sites
FDR: False discovery rate/ Adjusted-P value
GEO: Gene Expression Omnibus
DMGs: Differential DNA methylation genes
DEGs: Differential expression genes
KEGG: Kyoto Encyclopedia of Genes and Genomes
GO: Gene Ontology
FITC: Fluorescein isothiocyanate
Cy3: Cyanine 3
Cy5: Cyanine 5
DAPI: 4′ 6-Diamidino-2-phenylindole
5-mC: 5-Methylcytosine
Rb: Retinoblastoma protein
RNA-seq: RNA sequencing
scRNA-seq: Single-cell RNA sequencing

## References

[1] Mody MD, Rocco JW, Yom SS, Haddad RI, Saba NF (2021). Head and neck cancer. Lancet.

[2] Luo JJ, Young CD, Zhou HM, Wang XJ (2018). Mouse models for studying oral cancer: impact in the era of cancer immunotherapy. J Dent Res.

[3] Chang MS, Azin M, Demehri S (2022). Cutaneous squamous cell carcinoma: the frontier of cancer immunoprevention. Annu Rev Pathol.

[4] Kaidar-Person O, Gil Z, Billan S (2018). Precision medicine in head and neck cancer. Drug Resist Updat.

[5] Qi Z, Qiu Y, Wang Z, Zhang H, Lu L, Liu Y, Mathes D, Pomfret EA, Gao D, Lu SL, Wang Z (2021). A novel diphtheria toxin-based bivalent human EGF fusion toxin for treatment of head and neck squamous cell carcinoma. Mol Oncol.

[6] Redman JM, Friedman J, Robbins Y, Sievers C, Yang X, Lassoued W, et al. Enhanced neoepitope-specific immunity following neoadjuvant PD-L1 and TGF-beta blockade in HPV-unrelated head and neck cancer. J Clin Invest. 2022;132(18):e161400. 10.1172/JCI161400 PMC9479764 35727629

[7] Willey CD, Anderson JC, Trummell HQ, Naji F, de Wijn R, Yang ES, Bredel M, Thudi NK, Bonner JA (2019). Differential escape mechanisms in cetuximab-resistant head and neck cancer cells. Biochem Biophys Res Commun.

[8] Jiang Z, Lim SO, Yan M, Hsu JL, Yao J, Wei Y, et al. TYRO3 induces anti-PD-1/PD-L1 therapy resistance by limiting innate immunity and tumoral ferroptosis. J Clin Invest. 2021;131(8):e139434. 10.1172/JCI139434 PMC8262501 33855973

[9] Endicott JL, Nolte PA, Shen H, Laird PW (2022). Cell division drives DNA methylation loss in late-replicating domains in primary human cells. Nat Commun.

[10] Mori K, Hamada T, Beppu M, Tsuchihashi H, Goto Y, Kume K (2022). Detecting early-stage oral cancer from clinically diagnosed oral potentially malignant disorders by DNA methylation profile. Cancers (Basel).

[11] Calanca N, Francisco ALN, Bizinelli D, Kuasne H, Barros Filho MC, Flores BCT, Pinto CAL, Rainho CA, Soares MBP, Marchi FA (2023). DNA methylation-based depiction of the immune microenvironment and immune-associated long non-coding RNAs in oral cavity squamous cell carcinomas. Biomed Pharmacother.

[12] Carter B, Zhao K (2021). The epigenetic basis of cellular heterogeneity. Nat Rev Genet.

[13] Lyko F (2018). The DNA methyltransferase family: a versatile toolkit for epigenetic regulation. Nat Rev Genet.

[14] Zhang H, Gao Q, Tan S, You J, Lyu C, Zhang Y, Han M, Chen Z, Li J, Wang H (2019). SET8 prevents excessive DNA methylation by methylation-mediated degradation of UHRF1 and DNMT1. Nucleic Acids Res.

[15] Stankevicius V, Gibas P, Masiulionyte B, Gasiule L, Masevicius V, Klimasauskas S, Vilkaitis G (2022). Selective chemical tracking of Dnmt1 catalytic activity in live cells. Mol Cell.

[16] Estève PO, Chang Y, Samaranayake M, Upadhyay AK, Horton JR, Feehery GR, Cheng X, Pradhan S (2011). A methylation and phosphorylation switch between an adjacent lysine and serine determines human DNMT1 stability. Nat Struct Mol Biol.

[17] Di Ruscio A, Ebralidze AK, Benoukraf T, Amabile G, Goff LA, Terragni J, Figueroa ME, De Figueiredo Pontes LL, Alberich-Jorda M, Zhang P (2013). DNMT1-interacting RNAs block gene-specific DNA methylation. Nature.

[18] Meng W, Wu Y, He X, Liu C, Gao Q, Ge L, Wu L, Liu Y, Guo Y, Li X (2014). A systems biology approach identifies effective tumor-stroma common targets for oral squamous cell carcinoma. Cancer Res.

[19] Liu YY, Ding CZ, Chen JL, Wang ZS, Yang B, Wu XM (2022). A novel small molecular inhibitor of DNMT1 enhances the antitumor effect of radiofrequency ablation in lung squamous cell carcinoma cells. Front Pharmacol.

[20] Dongoran RA, Wang KH, Lin TJ, Yuan TC, Liu CH (2020). Anti-proliferative effect of statins is mediated by DNMT1 inhibition and p21 expression in OSCC cells. Cancers (Basel).

[21] Yang SC, Wang WY, Zhou JJ, Wu L, Zhang MJ, Yang QC, Deng WW, Sun ZJ (2022). Inhibition of DNMT1 potentiates antitumor immunity in oral squamous cell carcinoma. Int Immunopharmacol.

[22] Babar Q, Saeed A, Tabish TA, Pricl S, Townley H, Thorat N (2022). Novel epigenetic therapeutic strategies and targets in cancer. Biochim Biophys Acta Mol Basis Dis.

[23] Glaviano A, Foo ASC, Lam HY, Yap KCH, Jacot W, Jones RH, Eng H, Nair MG, Makvandi P, Geoerger B (2023). PI3K/AKT/mTOR signaling transduction pathway and targeted therapies in cancer. Mol Cancer.

[24] Wright NE, Mandal M, Clark MR (2023). Molecular mechanisms insulating proliferation from genotoxic stress in B lymphocytes. Trends Immunol.

[25] Galluzzi L, Vitale I, Aaronson SA, Abrams JM, Adam D, Agostinis P, Alnemri ES, Altucci L, Amelio I, Andrews DW (2018). Molecular mechanisms of cell death: recommendations of the nomenclature committee on cell death 2018. Cell Death Differ.

[26] Carneiro BA, El-Deiry WS (2020). Targeting apoptosis in cancer therapy. Nat Rev Clin Oncol.

[27] He Y, Sun MM, Zhang GG, Yang J, Chen KS, Xu WW, Li B (2021). Targeting PI3K/Akt signal transduction for cancer therapy. Signal Transduct Target Ther.

[28] Bury M, Le Calve B, Ferbeyre G, Blank V, Lessard F (2021). New Insights into CDK regulators: novel opportunities for cancer therapy. Trends Cell Biol.

[29] Mattei AL, Bailly N, Meissner A (2022). DNA methylation: a historical perspective. Trends Genet.

[30] Liu Y, Wu Y, Yang M, Yang J, Tong R, Zhao W, Wu F, Tian Y, Li X, Luo J, Zhou H (2023). Ionizing radiation-induced “zombie” carcinoma-associated fibroblasts with suppressed pro-radioresistance on OSCC cells. Oral Dis.

[31] Shi X, Luo J, Weigel KJ, Hall SC, Du D, Wu F, Rudolph MC, Zhou H, Young CD, Wang XJ (2021). Cancer-associated fibroblasts facilitate squamous cell carcinoma lung metastasis in mice by providing TGFbeta-mediated cancer stem cell niche. Front Cell Dev Biol.

[32] Luo J, Bian L, Blevins MA, Wang D, Liang C, Du D, Wu F, Holwerda B, Zhao R, Raben D (2019). Smad7 promotes healing of radiotherapy-induced oral mucositis without compromising oral cancer therapy in a xenograft mouse model. Clin Cancer Res.

[33] Wang Y, Bai X, Guo X, Gao X, Chen Y, Li H, Fan W, Han C (2022). Bioinformatics analysis combined with clinical sample screening reveals that leptin may be a biomarker of preeclampsia. Front Physiol.

[34] Liberzon A, Subramanian A, Pinchback R, Thorvaldsdottir H, Tamayo P, Mesirov JP (2011). Molecular signatures database (MSigDB) 3.0. Bioinformatics.

[35] Wang S, Wang X, Sun J, Yang J, Wu D, Wu F, Zhou H (2023). Down-regulation of DNA key protein-FEN1 inhibits OSCC growth by affecting immunosuppressive phenotypes via IFN-gamma/JAK/STAT-1. Int J Oral Sci.

[36] Choi JH, Lee BS, Jang JY, Lee YS, Kim HJ, Roh J, Shin YS, Woo HG, Kim CH (2023). Single-cell transcriptome profiling of the stepwise progression of head and neck cancer. Nat Commun.

[37] Gao R, Bai S, Henderson YC, Lin Y, Schalck A, Yan Y, Kumar T, Hu M, Sei E, Davis A (2021). Delineating copy number and clonal substructure in human tumors from single-cell transcriptomes. Nat Biotechnol.

[38] Qiu X, Mao Q, Tang Y, Wang L, Chawla R, Pliner HA, Trapnell C (2017). Reversed graph embedding resolves complex single-cell trajectories. Nat Methods.

[39] Pappalardi MB, Keenan K, Cockerill M, Kellner WA, Stowell A, Sherk C, Wong K, Pathuri S, Briand J, Steidel M (2021). Discovery of a first-in-class reversible DNMT1-selective inhibitor with improved tolerability and efficacy in acute myeloid leukemia. Nat Cancer.

[40] Azevedo Portilho N, Saini D, Hossain I, Sirois J, Moraes C, Pastor WA (2021). The DNMT1 inhibitor GSK-3484862 mediates global demethylation in murine embryonic stem cells. Epigenetics Chromatin.

[41] Van Tongelen A, Loriot A, De Smet C (2017). Oncogenic roles of DNA hypomethylation through the activation of cancer-germline genes. Cancer Lett.

[42] Chen Z, Zhang Y (2020). Role of Mammalian DNA Methyltransferases in Development. Annu Rev Biochem.

[43] Nishiyama A, Nakanishi M (2021). Navigating the DNA methylation landscape of cancer. Trends Genet.

[44] Arechederra M, Daian F, Yim A, Bazai SK, Richelme S, Dono R, Saurin AJ, Habermann BH, Maina F (2018). Hypermethylation of gene body CpG islands predicts high dosage of functional oncogenes in liver cancer. Nat Commun.

[45] Skvortsova K, Masle-Farquhar E, Luu PL, Song JZ, Qu W, Zotenko E, Gould CM, Du Q, Peters TJ, Colino-Sanguino Y (2019). DNA hypermethylation encroachment at CpG island borders in cancer is predisposed by H3K4 monomethylation patterns. Cancer Cell.

[46] Soulieres D, Faivre S, Mesia R, Remenar E, Li SH, Karpenko A, Dechaphunkul A, Ochsenreither S, Kiss LA, Lin JC (2017). Buparlisib and paclitaxel in patients with platinum-pretreated recurrent or metastatic squamous cell carcinoma of the head and neck (BERIL-1): a randomised, double-blind, placebo-controlled phase 2 trial. Lancet Oncol.

[47] Jimeno A, Shirai K, Choi M, Laskin J, Kochenderfer M, Spira A, Cline-Burkhardt V, Winquist E, Hausman D, Walker L, Cohen RB (2015). A randomized, phase II trial of cetuximab with or without PX-866, an irreversible oral phosphatidylinositol 3-kinase inhibitor, in patients with relapsed or metastatic head and neck squamous cell cancer. Ann Oncol.

[48] Tadesse S, Anshabo AT, Portman N, Lim E, Tilley W, Caldon CE, Wang S (2020). Targeting CDK2 in cancer: challenges and opportunities for therapy. Drug Discov Today.

[49] Yang C, Wang M, Gong Y, Deng M, Ling Y, Li Q, Wang J, Zhou Y (2023). Discovery and identification of a novel PI3K inhibitor with enhanced CDK2 inhibition for the treatment of triple negative breast cancer. Bioorg Chem.

[50] Fang G, Zhang P, Liu J, Zhang X, Zhu X, Li R, Wang H (2019). Inhibition of GSK-3β activity suppresses HCC malignant phenotype by inhibiting glycolysis via activating AMPK/mTOR signaling. Cancer Lett.

[51] Elgendy M, Cirò M, Hosseini A, Weiszmann J, Mazzarella L, Ferrari E, Cazzoli R, Curigliano G, DeCensi A, Bonanni B (2019). Combination of hypoglycemia and metformin impairs tumor metabolic plasticity and growth by modulating the PP2A-GSK3β-MCL-1 axis. Cancer Cell.

[52] Cross DA, Alessi DR, Cohen P, Andjelkovich M, Hemmings BA (1995). Inhibition of glycogen synthase kinase-3 by insulin mediated by protein kinase B. Nature.

[53] Jere SW, Houreld NN, Abrahamse H (2019). Role of the PI3K/AKT (mTOR and GSK3beta) signalling pathway and photobiomodulation in diabetic wound healing. Cytokine Growth Factor Rev.

[54] Hopkins BD, Pauli C, Du X, Wang DG, Li X, Wu D, Amadiume SC, Goncalves MD, Hodakoski C, Lundquist MR (2018). Suppression of insulin feedback enhances the efficacy of PI3K inhibitors. Nature.

[55] Kishikawa T, Higuchi H, Wang L, Panch N, Maymi V, Best S, et al. WWP1 inactivation enhances efficacy of PI3K inhibitors while suppressing their toxicities in breast cancer models. J Clin Invest. 2021;131(24):e140436. 10.1172/JCI140436 PMC8670846 34907909

[56] Chadwick JW, Macdonald R, Ali AA, Glogauer M, Magalhaes MA (2021). TNFalpha Signaling is increased in progressing oral potentially malignant disorders and regulates malignant transformation in an oral carcinogenesis model. Front Oncol.

[57] Chattopadhyaya S, Ghosal S (2022). DNA methylation: a saga of genome maintenance in hematological perspective. Hum Cell.

[58] Hoang NM, Rui L (2020). DNA methyltransferases in hematological malignancies. J Genet Genomics.

[59] Barcena-Varela M, Caruso S, Llerena S, Alvarez-Sola G, Uriarte I, Latasa MU, Urtasun R, Rebouissou S, Alvarez L, Jimenez M (2019). Dual Targeting of histone methyltransferase G9a and DNA-Methyltransferase 1 for the treatment of experimental hepatocellular carcinoma. Hepatology.

[60] Li Z, Li B, Yu H, Wang P, Wang W, Hou P, Li M, Chu S, Zheng J, Mao L, Bai J (2022). DNMT1-mediated epigenetic silencing of TRAF6 promotes prostate cancer tumorigenesis and metastasis by enhancing EZH2 stability. Oncogene.

[61] Liu H, Song Y, Qiu H, Liu Y, Luo K, Yi Y, Jiang G, Lu M, Zhang Z, Yin J (2020). Downregulation of FOXO3a by DNMT1 promotes breast cancer stem cell properties and tumorigenesis. Cell Death Differ.

[62] Xing J, Stewart DJ, Gu J, Lu C, Spitz MR, Wu X (2008). Expression of methylation-related genes is associated with overall survival in patients with non-small cell lung cancer. Br J Cancer.

[63] Ming X, Zhang Z, Zou Z, Lv C, Dong Q, He Q, Yi Y, Li Y, Wang H, Zhu B (2020). Kinetics and mechanisms of mitotic inheritance of DNA methylation and their roles in aging-associated methylome deterioration. Cell Res.

[64] Jones PA (2012). Functions of DNA methylation: islands, start sites, gene bodies and beyond. Nat Rev Genet.

[65] Yang Y, Ma S, Ye Z, Zheng Y, Zheng Z, Liu X, Zhou X (2023). Oncogenic DNA methyltransferase 1 activates the PI3K/AKT/mTOR signalling by blocking the binding of HSPB8 and BAG3 in melanoma. Epigenetics.

[66] Sun L, Zhao H, Xu Z, Liu Q, Liang Y, Wang L, Cai X, Zhang L, Hu L, Wang G, Zha X (2007). Phosphatidylinositol 3-kinase/protein kinase B pathway stabilizes DNA methyltransferase I protein and maintains DNA methylation. Cell Signal.

[67] Liu W, Gallego-Bartolome J, Zhou Y, Zhong Z, Wang M, Wongpalee SP, Gardiner J, Feng S, Kuo PH, Jacobsen SE (2021). Ectopic targeting of CG DNA methylation in Arabidopsis with the bacterial SssI methyltransferase. Nat Commun.

[68] Zhang J, Gan Y, Li H, Yin J, He X, Lin L, Xu S, Fang Z, Kim BW, Gao L (2022). Inhibition of the CDK2 and Cyclin A complex leads to autophagic degradation of CDK2 in cancer cells. Nat Commun.

[69] Gesbert F, Sellers WR, Signoretti S, Loda M, Griffin JD (2000). BCR/ABL regulates expression of the cyclin-dependent kinase inhibitor p27Kip1 through the phosphatidylinositol 3-Kinase/AKT pathway. J Biol Chem.

[70] Zhou BP, Liao Y, Xia W, Spohn B, Lee MH, Hung MC (2001). Cytoplasmic localization of p21Cip1/WAF1 by Akt-induced phosphorylation in HER-2/neu-overexpressing cells. Nat Cell Biol.

[71] Curtis M, Kenny HA, Ashcroft B, Mukherjee A, Johnson A, Zhang Y, Helou Y, Batlle R, Liu X, Gutierrez N (2019). Fibroblasts mobilize tumor cell glycogen to promote proliferation and metastasis. Cell Metab.

[72] Fan H, He Y, Xiang J, Zhou J, Wan X, You J, Du K, Li Y, Cui L, Wang Y (2022). ROS generation attenuates the anti-cancer effect of CPX on cervical cancer cells by inducing autophagy and inhibiting glycophagy. Redox Biol.

[73] Israelsen WJ, Dayton TL, Davidson SM, Fiske BP, Hosios AM, Bellinger G, Li J, Yu Y, Sasaki M, Horner JW (2013). PKM2 isoform-specific deletion reveals a differential requirement for pyruvate kinase in tumor cells. Cell.

[74] Pecoraro C, Faggion B, Balboni B, Carbone D, Peters GJ, Diana P, Assaraf YG, Giovannetti E (2021). GSK3beta as a novel promising target to overcome chemoresistance in pancreatic cancer. Drug Resist Updat.

[75] Mulholland DJ, Dedhar S, Wu H, Nelson CC (2006). PTEN and GSK3beta: key regulators of progression to androgen-independent prostate cancer. Oncogene.

