# Supplementary material for: DNMT1-targeting remodeling global DNA hypomethylation for enhanced tumor suppression and circumvented toxicity in oral squamous cell carcinoma
Source: Mol Cancer. 2024 May 16;23:104. doi: 10.1186/s12943-024-01993-1 (PMC11097543; doi:10.1186/s12943-024-01993-1)
Supplement: Supplementary file 1 — Supplementary Material 1. [file 12943_2024_1993_MOESM1_ESM.docx]

**Supplementary Materials**

**Table S1 Information of OSCC patients**

| Classification |  | N |
| --- | --- | --- |
| Gender | Male | 15 |
|  | Female | 7 |
| Age (years) | ＜40 | 1 |
|  | 40-65 | 12 |
|  | ≥65 | 9 |
| Pathology T stage | T2 | 9 |
|  | T3 | 4 |
|  | T4 | 9 |
| Pathology N stage | N0 | 13 |
|  | N1 | 4 |
|  | N2 | 4 |
|  | N3 | 1 |
| Pathology M stage | M0 | 22 |
|  | M1 | 0 |
| Histological grade | I | 12 |
|  | II | 8 |
|  | III | 2 |

**Table S2 Information of oral leukoplakia patients**

| Classification |  | N |
| --- | --- | --- |
| Gender | Male | 8 |
|  | Female | 5 |
| Age (years) | ＜40 | 1 |
|  | 40-65 | 8 |
|  | ≥65 | 4 |
| Pathological diagnosis | Oral leukoplakia | 13 |
| Degree of epithelial dysplasia | No (hyperplasia) | 6 |
|  | Mild | 5 |
|  | Severe | 2 |


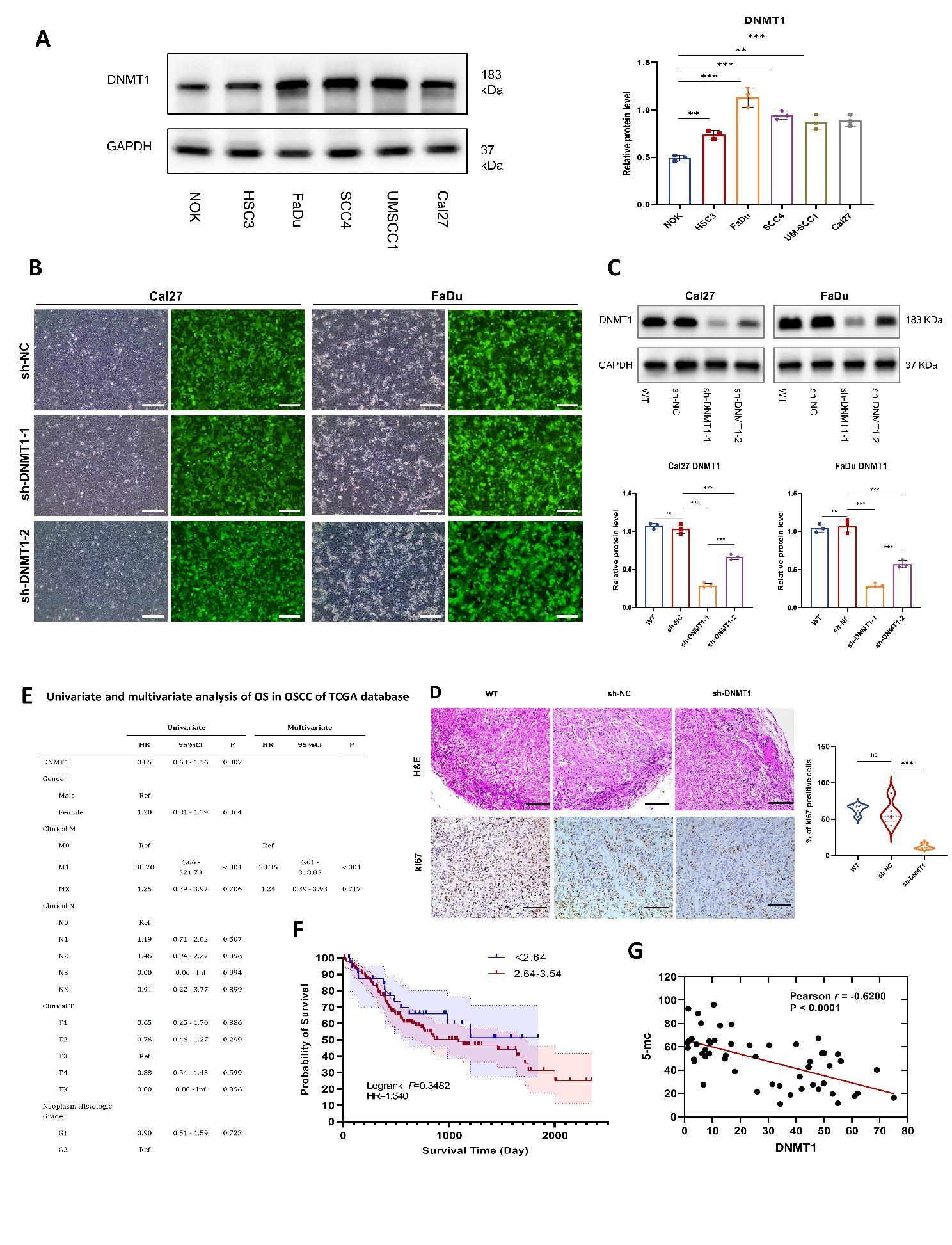


**Fig. S1** DNMT1 overexpression is common in several OSCC cell lines and its knockdown results in decreased cell proliferation. **a** Western blot analysis of DNMT1 in OSCC cells and NOK cells. **b** The GFP fluorescence expression of Cal27 and FaDu cells transfected by recombinant lentiviral virus of sh-DNMT1 after 72 hours. Scale bars, 200 μm. **c** Western blot analysis of DNMT1 expression in transfected and primary Cal27 and FaDu cells. **d** Representative images of H&E staining and IHC staining of Ki67 in xenograft OSCC tumors, as well as the quantification of Ki67. Scale bars, 100 μm. n=5 mice in each group. **P* < 0.05, ***P* < 0.01, and ****P* < 0.001 by unpaired Student’s *t* test. **e** Univariable and multivariable Cox regression of related factors in patients with OSCC. **f** Kaplan-Meier method estimated survival time in patients with OSCC stratified by DNMT1 expression level according to restricted cubic splines analysis. **g** Correlation analysis between the percentage of 5-mc positive cells and the DNMT1 expression level in oral human samples.


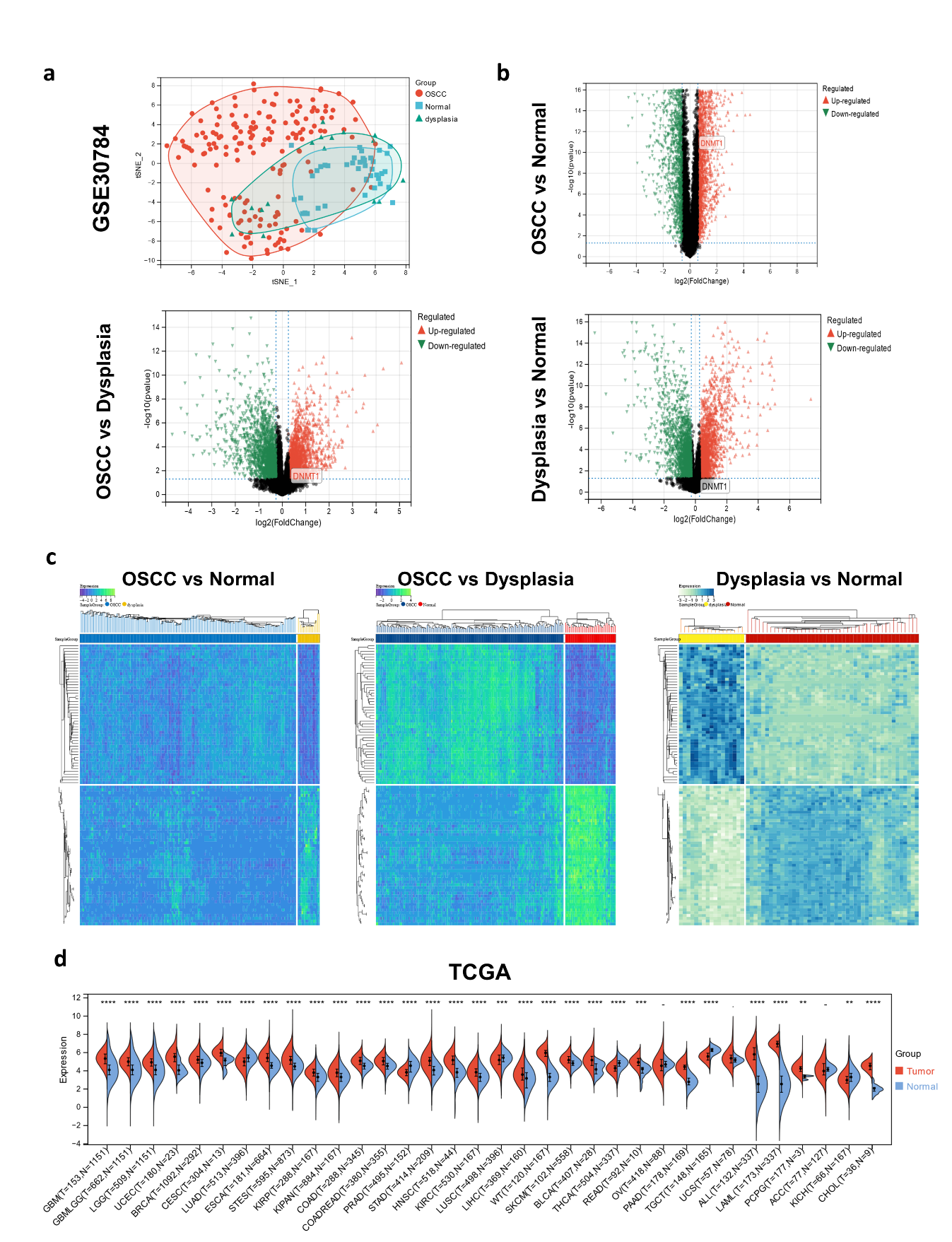


**Fig. S2** DNMT1 mRNA expression analysis based on GEO datasets and TCGA database. **a** Sample principal component analysis (PCA) after normalization of GSE30784 dataset. **b** Volcano plots showing all differentially expressed mRNA. **c** Heatmap showing Top 50 significantly up- and down- regulated DEGs of OSCC versus normal, OSCC versus dysplasia and dysplasia versus normal, respectively. **d** Pan-cancer analysis of DNMT1mRNA expression based on TCGA database.


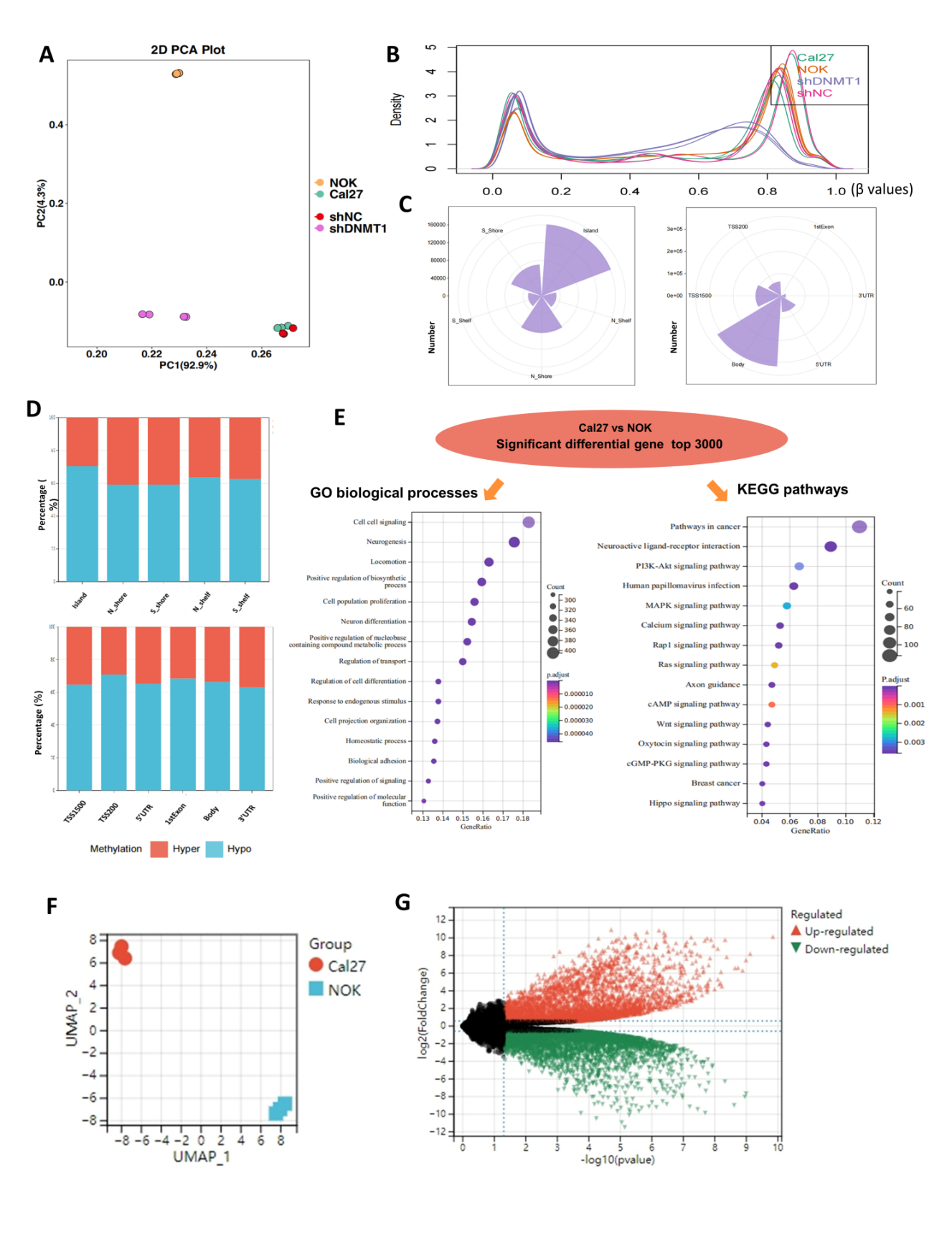


**Fig. S3** 850k chip analysis of OSCC cancer cells and NOK cells. **a** Sample PCA. **b** Ridgeline plot showing total normalized β value distribution of all samples. **c** Nightingale rose chart showing the probe distributions of all 850K methylation sites. **d** Histogram showing the proportion of all significantly DMSs with CpG island probe distribution and gene probe distribution respectively. **e** GO biological process and KEGG enrichment analysis of significant differential methylation genes Top 3000 between Cal27 and NOK cells. **f** and **g** The UMAP and volcano plot showed a remarkable difference between the Cal27 and NOK cells in RNAseq.


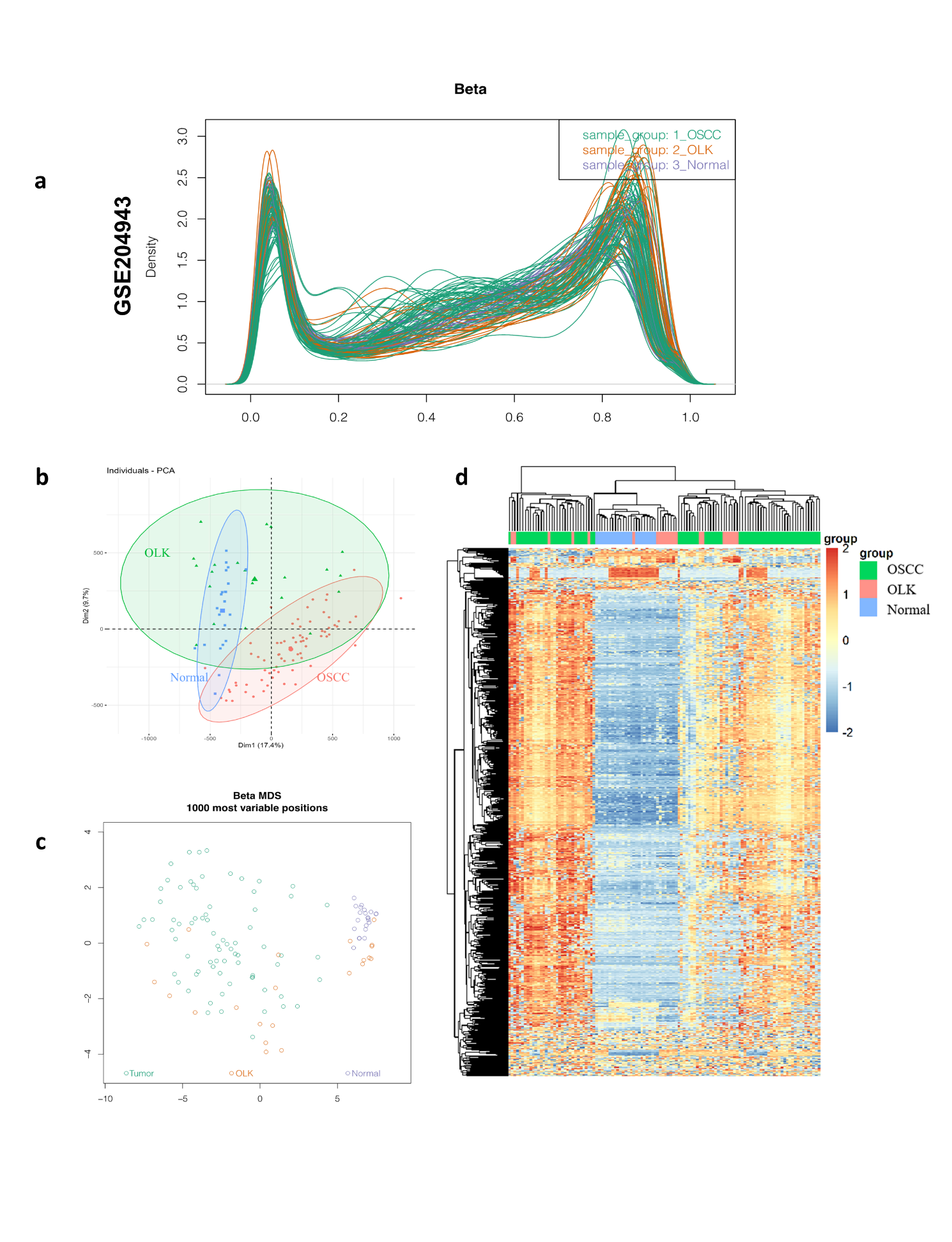


**Fig. S4** Data preprocessing for DNA methylation dataset GSE204943. **a** Ridgeline plot showing total normalized β value distribution of all samples. **b** Sample PCA after data normalization of all samples. **c** Multidimensional Scaling (MDS) analysis based on the Top 1000 differential DNA methylation sites with high variance value. **d** Heatmap showing values of 1000 most differential DNA methylation sites among samples.


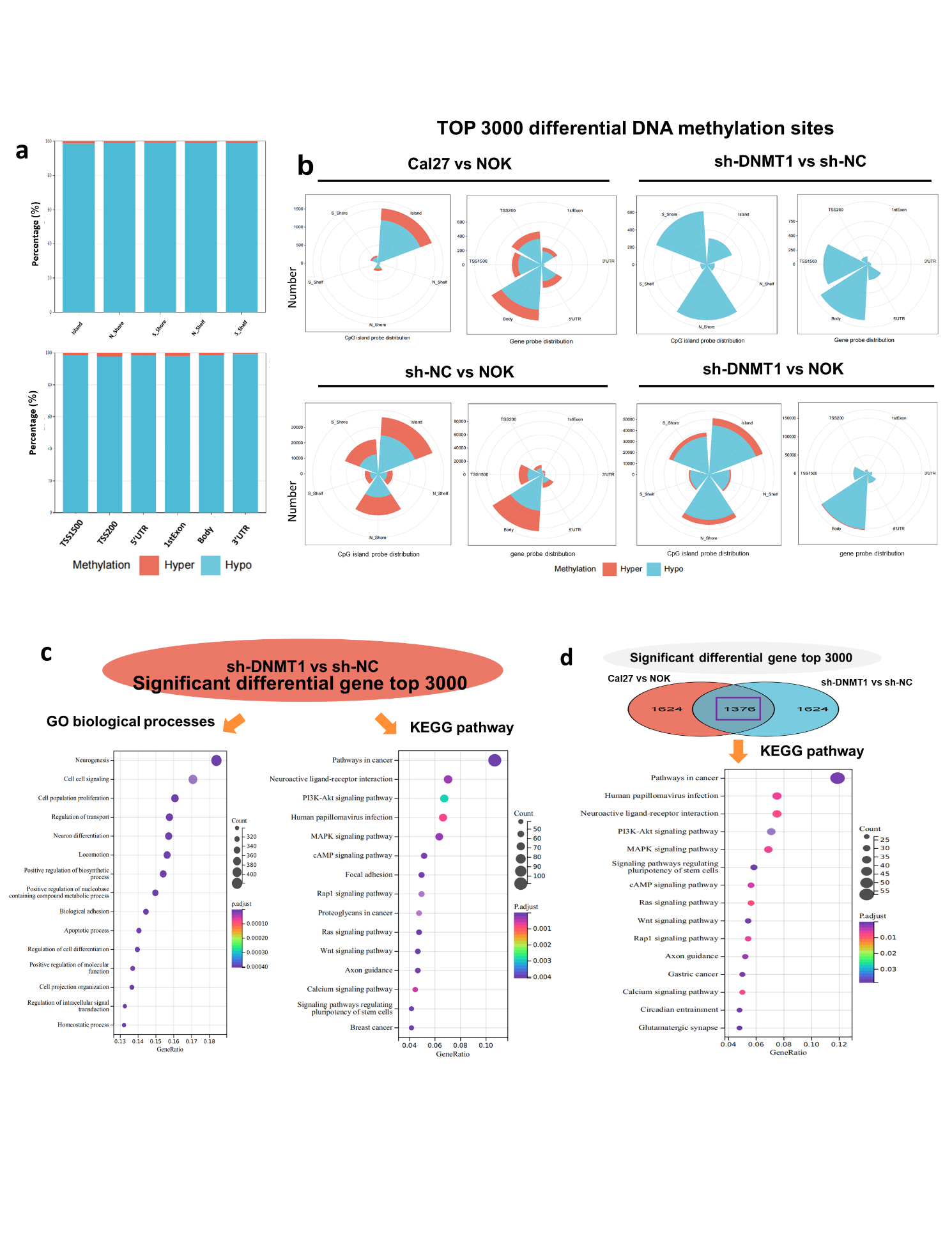


**Fig. S5** DNMT1 targeting remodeled the global DNA methylation of cancer cells and linked to PI3K-AKT pathways. **a** Histogram showing the proportion of all significantly DMSs with CpG island probe distribution and gene probe distribution respectively. **b** Nightingale rose chart showing the number of significant DMSs Top 3000 according to CpG island probe distribution and gene probe distribution among different cell types. **c** GO biological process and KEGG enrichment analysis of significant DMEs Top 3000 between sh-NC and sh-DNMT1 cancer cells. **d** KEGG enrichment analysis of DMEs overlapped from Top 3000 screened out of Cal27 versus NOK cells and sh-DNMT1 versus sh-NC cancer cells.


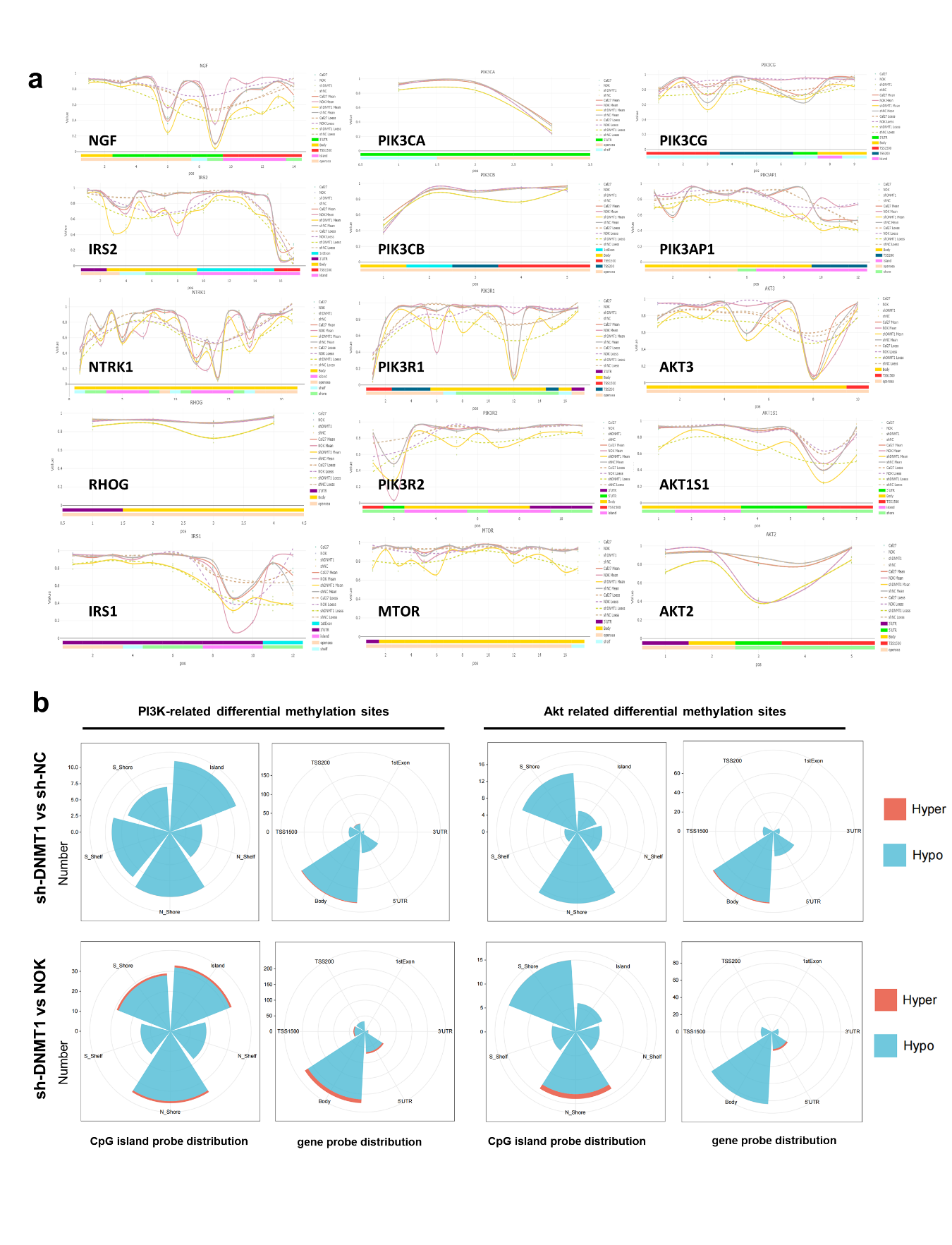


**Fig. S6** PI3K-AKT signaling pathway were validated as a critical downstream pathway of DNMT1- dependent DNA hypomethylation pattern. **a** The significantly DMSs enriched in key genes in activating PI3K-AKT pathway. Solid lines, the mean β values of each cell line; dotted lines, the β values loess of each cell lines. The genes were listed as NGF, PIK3CA, PIK3CG, IRS2, PIK3CB, PIK3AP1, NTRK1, PIK3R1, AKT3, RHOA, PIK3R2, AKT1S1, IRS1, MTOR and AKT2. **b** Nightingale rose chart showing the number of all significant DMSs associated with PI3K- AKT pathways according to CpG island probe distribution and gene probe distribution, in sh-DNMT1 cancer cells compared to sh-NC cancer cells and NOK cells.


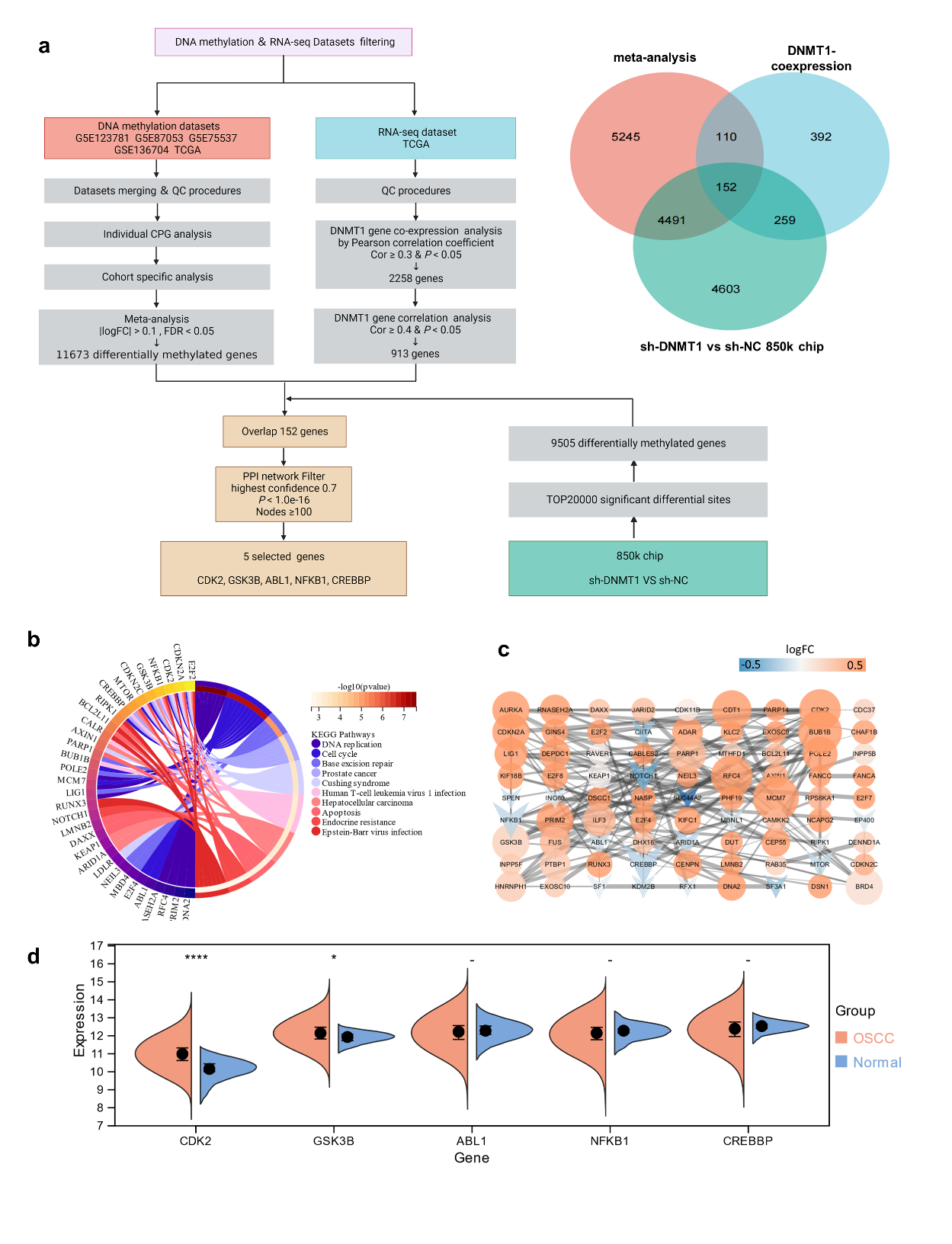


**Fig. S7** CDK2 and GSK3β were screened out as the key functional genes downstream to DNMT1-related DNA methylation alterations. **a** The workflow to obtain 5 out of 152 overlapped DEGs closely to DNMT1-related DNA methylation alterations. **b** Circle graph indicated the KEGG enrichment of the 152 DEGs, with ranking pathways of DNA replication, cell cycle, and base excision repair. **c** The PPI network analysis and visualization , annotate the node shape with the results of RNA differential expression in OSCC sets from the TCGA database, with circles representing up-regulation and inverted triangles representing down-regulation; annotate the color change according to the logFC value; the size of the node represents the number of interacting proteins, the larger the node meaning the more interacting proteins; the width of edge represents the interaction score of the proteins, the thicker the edge meaning the higher interaction score. **d** The mRNA expression of the 5 genes in OSCC compared to normal oral tissues based on TCGA database.
