# Supplementary figures and images for: DNMT1-targeting remodeling global DNA hypomethylation for enhanced tumor suppression and circumvented toxicity in oral squamous cell carcinoma
Source: Mol Cancer. 2024 May 16;23:104. doi: 10.1186/s12943-024-01993-1 (PMC11097543; doi:10.1186/s12943-024-01993-1)

**Original and uncropped Blots images**


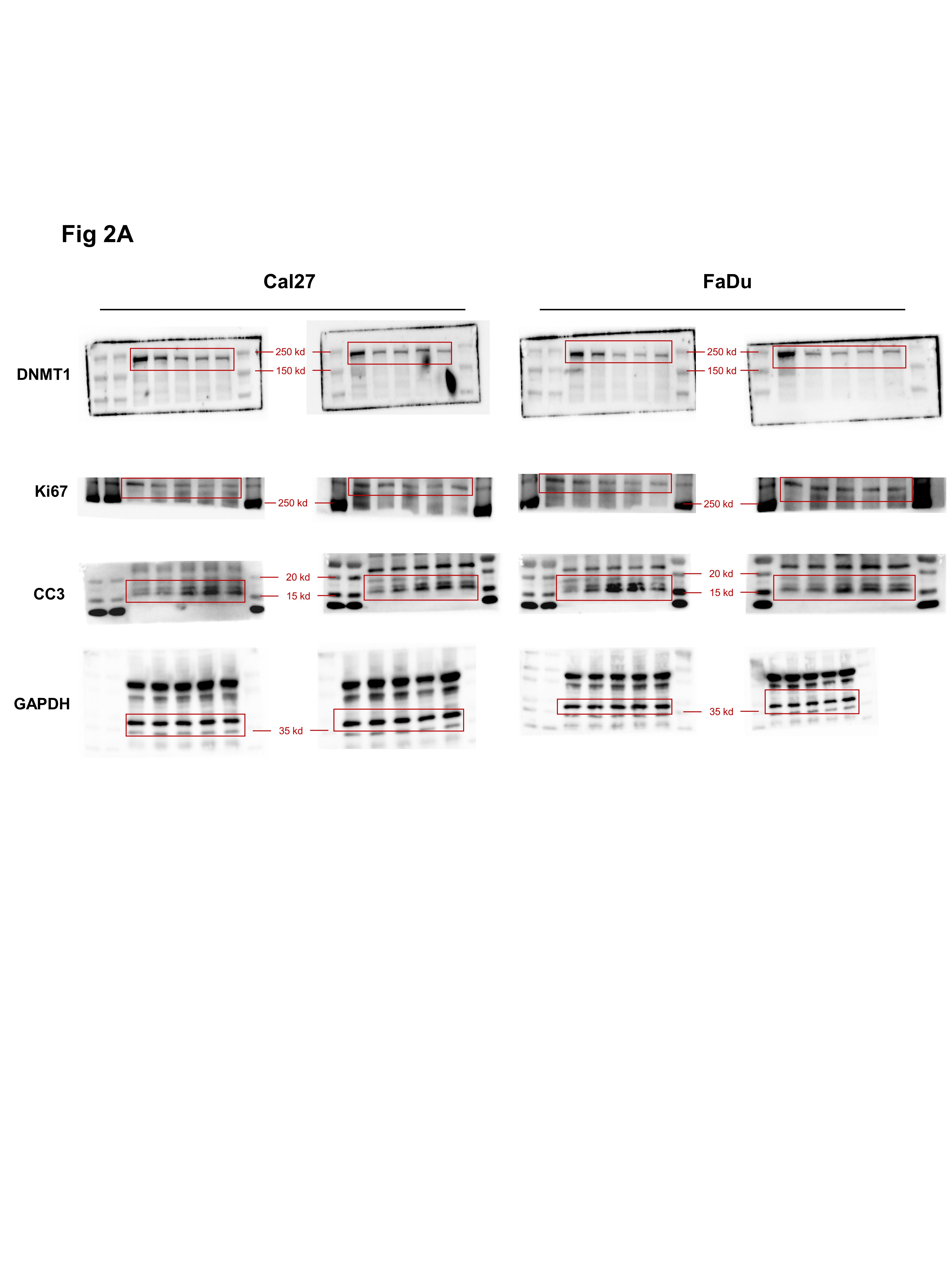

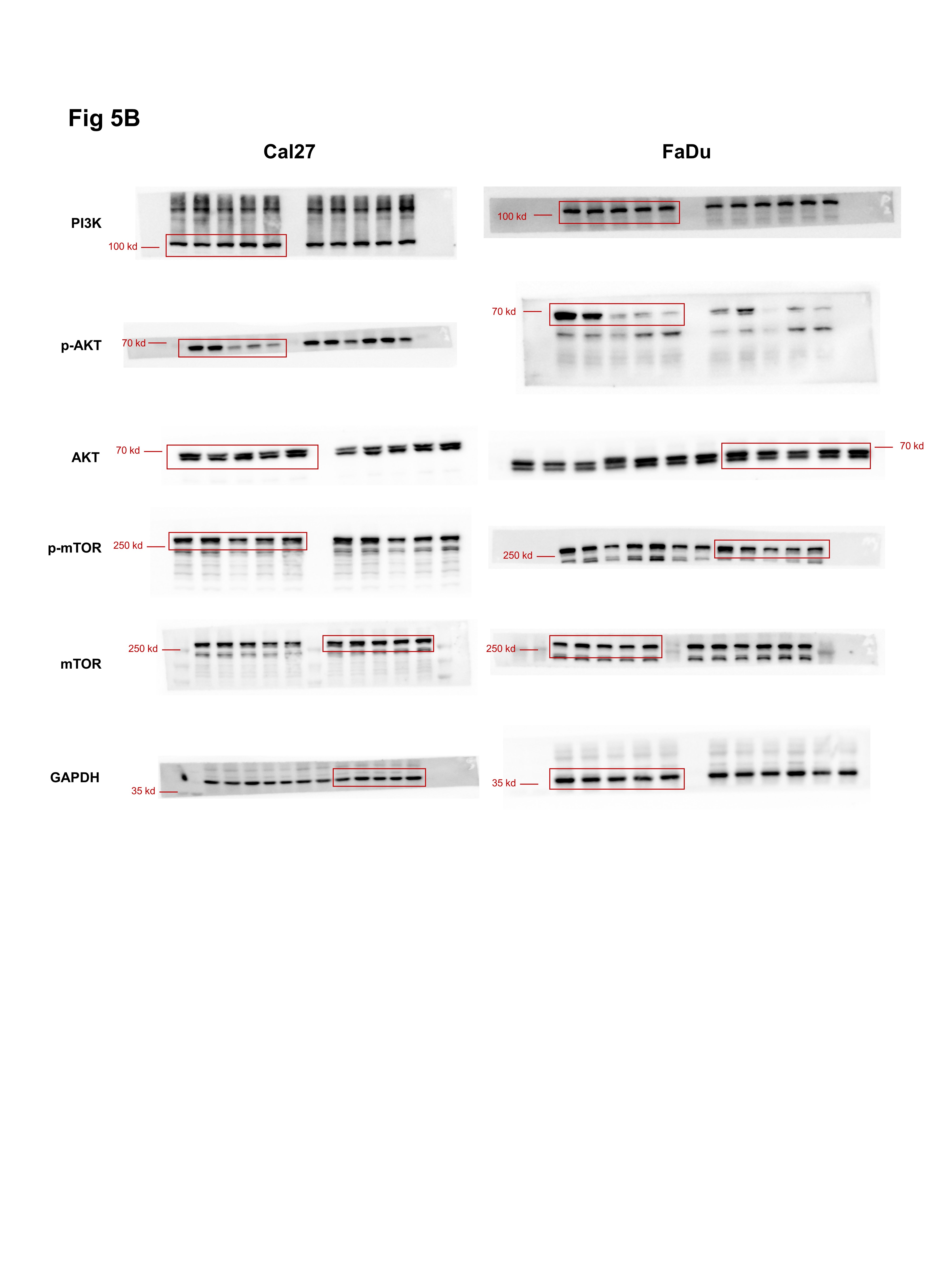

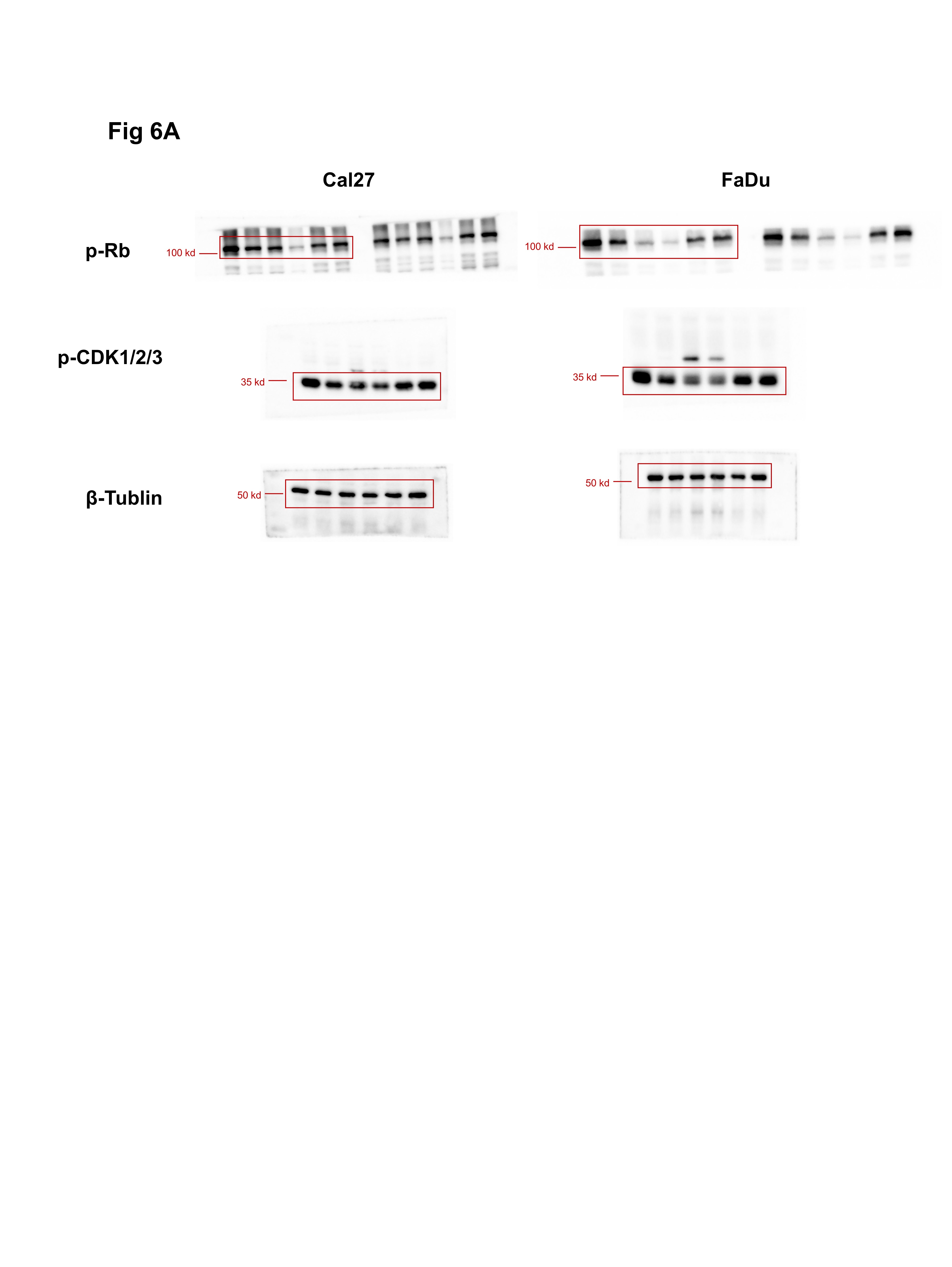


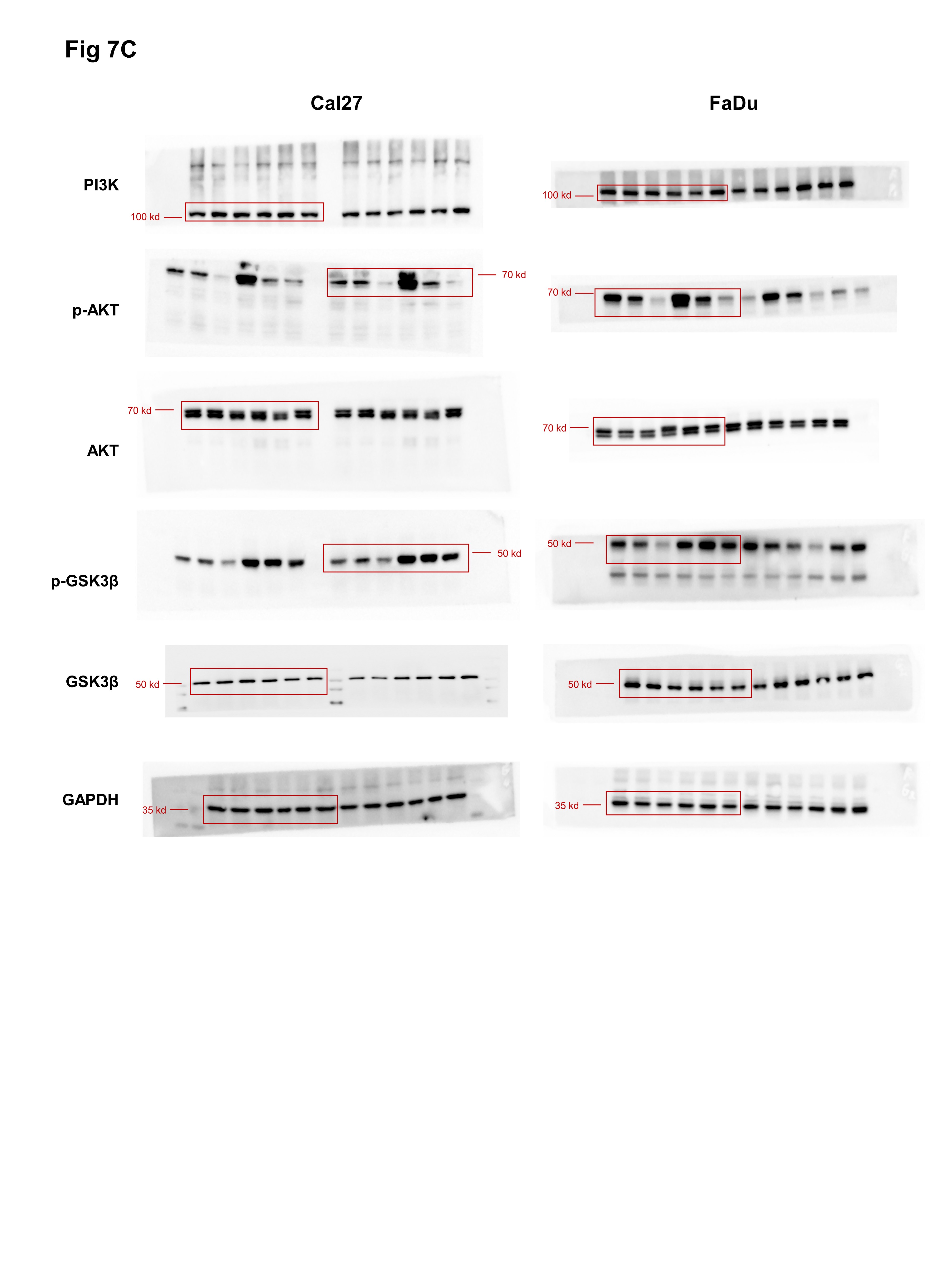


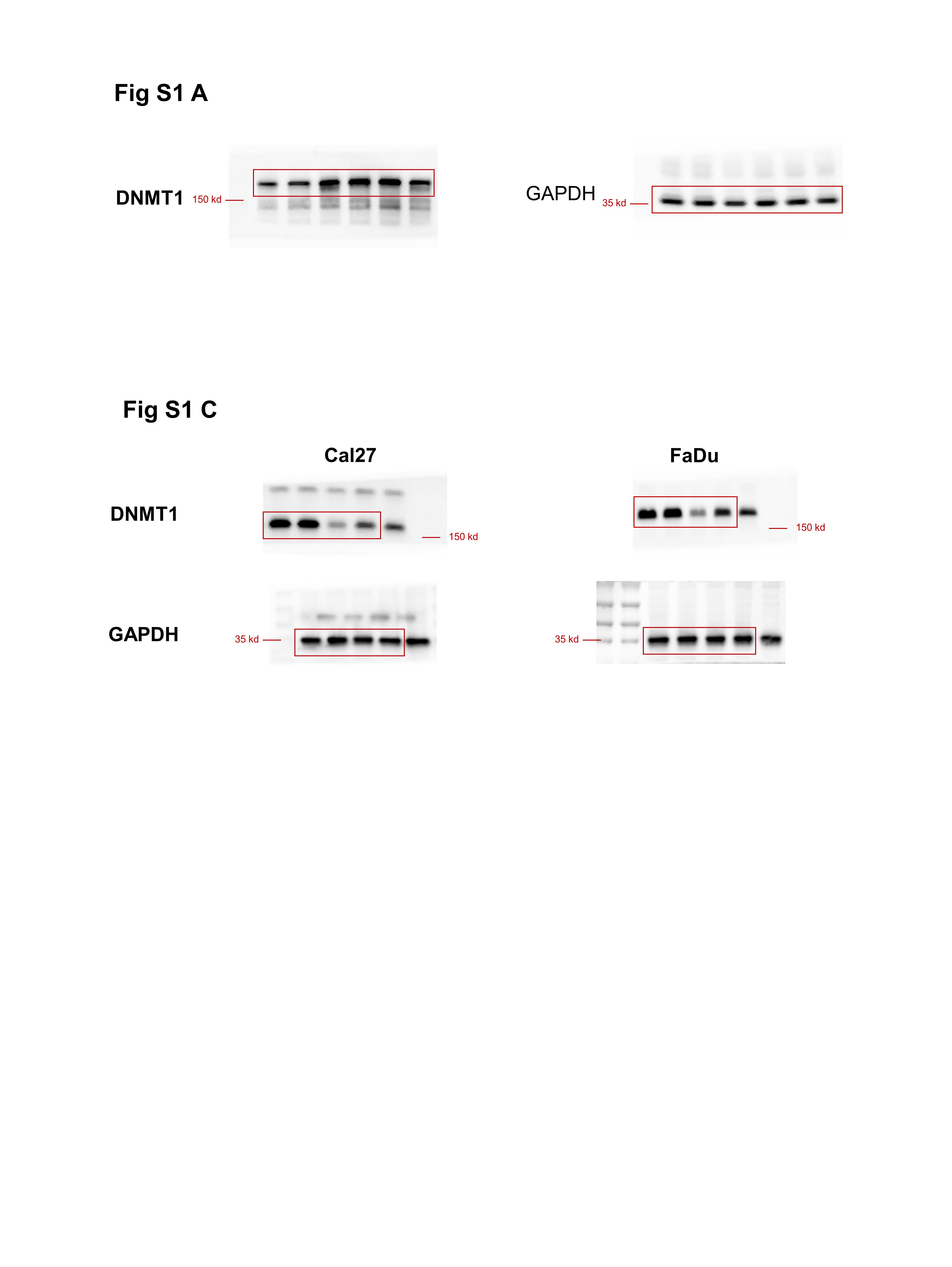

Supplement: Supplementary file 2 — Supplementary Material 2. [file 12943_2024_1993_MOESM2_ESM.docx]
